# Self-Administered Hypnosis vs Sham Hypnosis for Hot Flashes: A Randomized Clinical Trial

**DOI:** 10.1001/jamanetworkopen.2025.42537

**Published:** 2025-11-11

**Authors:** Gary Elkins, Noel Arring, Grant Morgan, Tierney Lorenz, Vanessa Muniz, Carrie Lafferty, Katherine Scheffrahn, Cameron Alldredge, Debra Barton

**Affiliations:** 1 Department of Psychology and Neuroscience, Baylor University, Waco, Texas; 2 College of Nursing, University of Tennessee Knoxville, Knoxville; 3 Department of Educational Psychology, Baylor University, Waco, Texas; 4 Department of Psychology, University of Nebraska–Lincoln, Lincoln

## Abstract

**Importance:**

Many women experience significant adverse effects from menopausal hot flashes. Clinical hypnosis has been shown to be an effective and safe nonhormonal treatment for hot flashes, but there is a need to increase the accessibility of this intervention.

**Objectives:**

To evaluate the efficacy of a self-administered hypnosis intervention for hot flashes compared with a self-administered sham white noise hypnosis control, evaluate the efficacy of the intervention compared with sham hypnosis for hot flash activity interference and perception of benefit, and examine the interaction between practice adherence and treatment effect.

**Design, Setting, and Participants:**

This multicenter, 2-arm, single-blind, randomized clinical trial was conducted from March 4, 2019, to February 16, 2024. Participants were 250 postmenopausal women self-reporting a minimum of 4 daily or 28 weekly hot flashes at baseline who were recruited at Baylor University in Waco, Texas, and the University of Michigan in Ann Arbor.

**Intervention:**

Participants were randomized into either a 6-week self-administered hypnosis condition or a self-administered sham white noise condition. The hypnotic inductions consisted of cooling imagery and suggestions for relaxation, while the white noise condition was designed to be an active control accounting for all components present in the intervention arm, including placebo and cognitive expectancy effects, without the therapeutic components present in the active arm.

**Main Outcomes and Measures:**

The primary outcome was the change in hot flash score from baseline to 6 weeks, with a 12-week follow-up. The hot flash score (lower scores indicate less frequent and less severe hot flashes) was calculated as a product of the frequency and severity of hot flashes as reported in their daily hot flash diary. Secondary outcomes included hot flash activity interference, measured using the Hot Flash Related Daily Interference Scale (HFRDIS; score range, 0-100; lower scores indicate less interference from hot flashes on participants’ daily activities, enjoyment, or quality of life), and perception of benefit, measured using the Subject Global Impression of Change. Analysis was conducted on an intent-to-treat basis.

**Results:**

Participants were 250 women (mean [SD] age, 55.9 [6.9] years) experiencing hot flashes, 62 (24.8%) of whom had a history of breast cancer. Overall improvement in mean (SD) hot flash scores at 6 weeks was greater for participants in the hypnosis group (baseline score, 88.7 [61.3] vs 6-week score, 41.3 [50.8]; 53.4% decrease) than those in the white noise group (baseline score, 94.6 [81.6] vs 6-week score, 55.9 [50.9]; 40.9% decrease; *P* = .04). The intervention group reported a significantly greater reduction in daily interference from baseline to week 6 (baseline mean [SD] HFRDIS score, 49.3 [22.6] vs 6-week score, 25.0 [22.4]; 49.3% decrease) compared with the control group (baseline mean [SD] HFRDIS score, 47.3 [22.4] vs 6-week score, 29.6 [22.0]; 37.4% decrease) as well as greater perceived benefits (90.3% [93 of 103] vs 64.3% [63 of 98]).

**Conclusions and Relevance:**

In this randomized clinical trial, the clinical hypnosis group experienced significantly greater reductions of hot flash scores and daily interference from hot flashes compared with the active control condition at week 6. This study suggests that hypnosis delivered through self-administered audio files is a clinically significant and effective method to reduce hot flashes in postmenopausal women.

**Trial Registration:**

ClinicalTrials.gov Identifier: NCT03572153

## Key Points

**Question:**

Does a self-administered clinical hypnosis intervention effectively reduce hot flashes compared with a sham hypnosis?

**Findings:**

In this randomized clinical trial of 250 postmenopausal women, the hypnosis group experienced a significantly greater reduction in hot flash scores (frequency × severity) compared with the control group at week 6 (53.4% vs 40.9%). The intervention group reported a significantly greater reduction in daily interference from hot flashes (49.3% vs 37.4%) from baseline to week 6 and greater perceived benefits (90.3% vs 64.3%) compared with the sham hypnosis group.

**Meaning:**

This study suggests that self-administered clinical hypnosis is an effective treatment option for reducing hot flash frequency and severity by over 50%.

## Teaser

This randomized clinical trial evaluates the efficacy of a self-administered hypnosis intervention for hot flashes compared with a self-administered sham hypnosis control and evaluates the efficacy of the intervention for hot flash activity interference and perception of benefit.

## Introduction

Hot flashes can cause sweating, discomfort, anxiety, fatigue, and sleep interference, leading to adverse health outcomes and decreased quality of life.^1,2,3,4,5,6^ Up to 80% of women in the general population report hot flashes from the menopause transition and beyond, persisting on average for 4 to 7 years.^7,8,9^ Although hormone therapy is effective in treating hot flashes, its use is contraindicated for individuals initiating hormone therapy more than 10 years from menopause onset or older than 60 years, and with a history of breast cancer, uterine cancer, thromboembolic, or cardiovascular diseases.^10,11,12^ Therefore, options for nonhormonal treatments of hot flashes are important.^13,14,15,16,17,18,19,20,21,22,23,24,25,26^

In its latest position statement, the Menopause Society recommends the use of clinical hypnosis for the treatment of vasomotor symptoms based on level 1 evidence.^27^ Randomized clinical trials of therapist-delivered hypnosis have shown clinically significant efficacy for reductions in the frequency and severity of hot flashes.^28,29,30^ In prior studies assessing the effectiveness of clinical hypnosis across different delivery modes, self-administered hypnosis interventions for irritable bowel syndrome and chronic pain have shown promising results with effectiveness comparable to in-person delivery,^31^ while also improving accessibility.^32^ However, to our knowledge, no prior study has examined self-administered hypnosis for hot flashes.

This randomized clinical trial evaluates the efficacy of a self-administered hypnotic intervention for hot flashes that uses an innovative active control condition of sham hypnosis rather than an inert or waiting list control. This study’s primary objective was to fully evaluate the efficacy of the self-administered hypnosis intervention for hot flashes when compared with a sham hypnosis control. Individuals with a history of breast cancer may experience more severe hot flashes due to the sudden onset of symptoms after cancer treatment, as opposed to the gradual transition seen in natural menopause.^33,34,35^ This study presents a subgroup analysis of the primary outcomes for participants with a history of breast cancer. The secondary objectives were to evaluate the efficacy of self-administered hypnosis compared with sham hypnosis for hot flash daily interference and perception of benefit and to examine the interaction between practice adherence and treatment effect.

## Methods

### Design, Setting, and Participants

This study was a 2-arm, randomized clinical trial. Recruitment and data collection were completed at Baylor University, Waco, Texas, and the University of Michigan at Ann Arbor. The Baylor University institutional review board approved the study protocol with the included statistical analysis plan (Supplement 1). The results reported in this study adhere to the Consolidated Standards of Reporting Trials (CONSORT) reporting guideline for randomized clinical trials. All participants in the study provided written informed consent.

Participants were recruited from March 4, 2019, to February 6, 2024, when accrual goals were completed. Due to the COVID-19 pandemic, there was a brief hiatus during early 2020 while recruitment was adjusted to be conducted virtually; however, there was no change in the intervention’s delivery. Eligible participants met with a study research assistant on 2 occasions (at baseline and at randomization) either in person or virtually. Participants were contacted by telephone once weekly for 5 weeks during their intervention period and once prior to their week 12 follow-up to remind them of data collection. A schedule of activities is provided in eTable 1 in Supplement 2.

Prior to the study commencing, the target sample size was determined using an a priori power calculation to detect a moderately strong differential (ie, interaction) effect hot flash reduction with a moderation effect in a mixed-effects analysis of variance (ANOVA) model. Eligible participants were postmenopausal women self-reporting a minimum of 4 daily hot flashes or 28 weekly hot flashes at baseline. Participants were asked to discontinue other putative therapies for hot flashes for at least 1 month prior to enrollment (except vitamin E). Women with a diagnosis of ductal carcinoma in situ or invasive breast cancer stages 0 to III currently receiving endocrine therapy, or taking antidepressants, were allowed to participate in the study if their treatment remained consistent throughout the duration of the study. Exclusion criteria included currently using hypnosis for any condition, receiving other simultaneous treatment for vasomotor symptoms (except antidepressants), a diagnosis of stage IV breast cancer, severe psychological illness in the past 5 years, a 4-item Patient Health Questionnaire score of 9 or more, or being a non-English speaker.

### Blinding and Randomization

The primary investigators, data collectors, and study statistician (who made the allocation sequence) remained blinded to group allocation until the database was locked, deidentified, and ready for analysis. Participants were blinded to the study hypotheses to avoid bias regarding which arm was the experimental treatment. Study interventionists enrolled and assigned participants to interventions.

Enrolled participants were randomized to either self-administered hypnosis or self-administered sham hypnosis. Randomization was accomplished using stratified permuted block randomization with fixed blocks of size 4. Two strata were used: site (Baylor University or University of Michigan) and participant subgroup (postmenopausal or breast cancer).

### Intervention

Participants in the hypnosis intervention were given educational material on the use of hypnosis for the treatment of hot flashes and were asked to listen to daily 20-minute audio-recorded hypnosis sessions for 6 weeks. The audio recordings included hypnotic relaxation induction and mental imagery for coolness.

The control arm sham hypnosis intervention consisted of white noise audio recordings labeled as “hypnosis” and educational material about the use of the intervention for hot flashes. Participants in the control arm were asked to listen to white noise audio recordings, time matched to the audio recordings of the self-hypnosis intervention arm, every day throughout weeks 2 to 6.

### Primary Outcomes

Participants completed a Hot Flash Daily Diary (HFD)^36^ to measure the daily frequency and severity of hot flashes during their participation in the study. The HFD is a validated measure where participants mark each time they experience a hot flash each day for a week, based on different severity levels (mild, moderate, severe, and very severe).^28,36,37^ A HFD was collected at baseline to determine eligibility. Six HFDs were collected at the end of the intervention period, and 1 HFD was collected at follow-up (week 12). A total hot flash score was calculated at these 3 time points for each participant by multiplying the participants’ frequency and severity ratings of hot flashes, divided by the total number of days. A lower score indicates less frequent and less severe hot flashes.

### Secondary Outcomes

Hot flash activity interference was measured using the Hot Flash Related Daily Interference Scale (HFRDIS).^38^ Participants rated the degree (0-10) to which their hot flashes interfered with various daily activities and overall quality of life (total score range, 0-100; lower scores indicate less interference from hot flashes on participants’ daily activities, enjoyment, or quality of life). Perception of benefit was measured at the end point using the Subject’s Global Impression of Change scale,^39,40^ which is a 7-point scale, where lower scores indicate a greater perceived benefit. Participants rated the change in hot flashes since beginning the study (“very much better” to “very much worse”). Hot flash interference and perception of benefit were measured at baseline, end point (week 6), and follow-up (week 12) via questionnaires distributed to the participants. The Self-Hypnosis Practice Log was distributed to all participants to complete daily throughout the intervention period and daily during week 12 as a measure of intervention adherence.^41^ Participants submitted their practice logs detailing adherence at the end of each week during the intervention period, and they submitted their follow-up practice log at the end of week 12. In addition, baseline demographic data (marital status, employment status, educational level, history of breast cancer, and race and ethnicity [Asian, Black or African American, Hispanic or Latina, Native Hawaiian or Pacific Islander, White, or other (did not specify a race or ethnicity) or >1 race]) were collected via self-report, used to ensure there was no systematic bias present across participant characteristics.

### Adverse Events

At each weekly check-in, interventionists asked participants to report any adverse events, defined as any unfavorable and unintended diagnosis, symptom, syndrome, or disease occurring during the study. Adverse events were recorded regardless of their association with the study. Reports of adverse events were reviewed by an independent data safety monitoring board.

### Statistical Analysis

We conducted an intent-to-treat analysis. Participants were analyzed based on their assigned groups. All continuous variables were examined graphically to understand their distributional characteristics. For descriptive analyses, mean (SD) values were generated for continuous variables, and frequency distributions were generated for categorical variables. The primary inferential procedure used for hot flash scores and severity levels was a mixed-effects ANOVA model, where time (ie, baseline, week 6, and week 12) was a within-individuals factor and intervention group was a between-individuals factor. The same analyses were conducted with a subset of patients with a history of breast cancer.

For all mixed-effects ANOVA models, the preestablished maximum type I error rate was 5%, and η^2^ was used as the effect size estimate. Where there are violations, sphericity is noted, Huynh-Feldt corrections were used for greater type I error control. Mean weekly self-reported adherence scores were calculated and used in an interaction analysis. The mean percentage improvement was computed for each group, and the distributional differences between treatment conditions were calculated with the χ^2^ test with the Cramer *V* as the effect size estimate.

Patterns of missing data on the hot flash scores were examined. At each of the data collection points—baseline, week 6, and week 12—there was, respectively, 95.2% (238 of 250), 82.0% (205 of 250), and 76.4% (191 of 250) completion. Most participants (74.4% [186 of 250]) had complete data across all 3 data collection points. Also, 12.8% of participants (32 of 250) had hot flash scores for baseline only, and 6.8% (17 of 250) had hot flash scores for baseline and week 6 only. The original data were analyzed as described, and 50 datasets were also imputed with complete data on the primary outcome variables using the Markov chain Monte Carlo simulation. The output from the original analyses and summary of imputed datasets for the primary outcome variable are reported. There were no discrepancies between the analyses from the original data and the imputed data.

The type I error rate was set at 5%. Omnibus ANOVA tests were 1-sided; all other tests were 2-sided. Analysis was performed with SPSS, version 29.0.2.0 (IBM Corp).

## Results

### Demographic Characteristics

**Figure 1.**
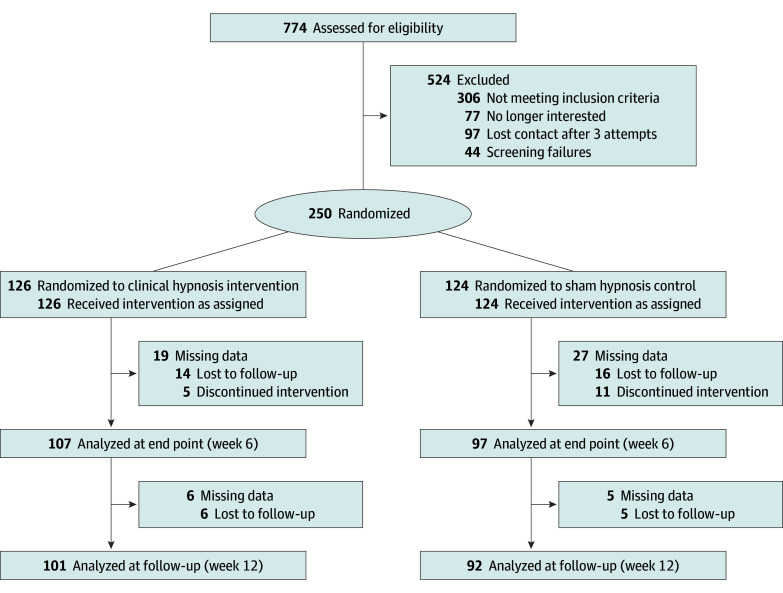
Flowchart of Participants This flowchart represents the process of accrual, randomization, and participation in the study.

From 774 individuals assessed for eligibility, the final analytic sample consisted of 250 women (mean [SD] age, 55.9 [6.9] years; 7 Asian participants [2.8%], 39 Black or African American participants [15.7%], 15 Hispanic or Latina participants [6.0%], 1 Native Hawaiian or Pacific Islander participant [0.4%], 187 White participants [75.1%], and 13 participants of other race or >1 race [5.2%]) (Table 1). The trial ended when the target sample was achieved. Of those 250 participants, 126 (50.4%) were randomized into a self-administered hypnosis condition, and 124 (49.6%) were randomized into a sham hypnosis control condition (Figure 1). Sixty-two participants (24.8%; 33 in the intervention arm and 29 in the control arm) reported a history of breast cancer. Full demographic characteristics are provided in Table 1.

**Table 1.** Full Demographic Characteristics of Analytic Sample Participants

Variable	Study arm, No. (%)		
	Hypnosis (n = 126)	Sham hypnosis (n = 124)	Total (N = 250)
Race and ethnicity			
Asian	5 (4.0)	2 (1.6)	7 (2.8)
Black or African American	19 (15.1)	20 (16.1)	39 (15.6)
Hispanic or Latina			
No	118 (93.7)	115 (92.7)	233 (93.2)
Yes	7 (5.6)	8 (6.5)	15 (6.4)
Did not report	1 (0.8)	1 (0.8)	2 (0.8)
Native Hawaiian or Pacific Islander	0	1 (0.8)	1 (0.4)
White	96 (76.2)	91 (73.4)	187 (74.8)
Other or >1 race^a^	6 (4.8)	7 (5.6)	13 (5.2)
Did not report	0	1 (0.8)	1 (0.4)
Marital status			
Married	99 (78.6)	89 (71.8)	188 (75.2)
Divorced	17 (13.5)	19 (15.3)	36 (14.4)
Single, never married	6 (4.8)	7 (5.6)	13 (5.2)
Single, living with partner	1 (0.8)	3 (2.4)	4 (1.6)
Widowed	1 (0.8)	1 (0.8)	2 (0.8)
Other	1 (0.8)	1 (0.8)	3 (1.2)
Did not report	1 (0.8)	3 (2.4)	4 (1.6)
Employment status			
Employed, full time	74 (58.7)	63 (50.8)	137 (54.8)
Retired	18 (14.3)	24 (19.4)	42 (16.8)
Employed, part time	16 (12.7)	12 (9.7)	28 (11.2)
Homemaker	11 (8.7)	11 (8.9)	22 (8.8)
Other	2 (1.6)	8 (6.5)	10 (4.0)
Unemployed	5 (4.0)	3 (2.4)	8 (3.2)
Did not report	0	3 (2.4)	3 (1.2)
Educational level			
High school or GED certification	10 (7.9)	10 (8.1)	20 (8.0)
Some college	24 (19.0)	16 (12.9)	40 (16.0)
Associate’s degree	16 (12.7)	16 (12.9)	32 (12.8)
Bachelor’s degree	38 (30.2)	46 (37.1)	84 (33.6)
Master’s degree	35 (27.8)	24 (19.4)	59 (23.6)
Doctoral degree	3 (2.4)	11 (8.9)	14 (5.6)
Did not report	0	1 (0.8)	1 (0.4)
History of breast cancer			
Yes	33 (26.2)	29 (23.4)	62 (24.8)
No	92 (73.0)	89 (71.8)	181 (72.4)
Did not report	1 (0.8)	6 (4.8)	7 (2.8)

Abbreviation: GED, General Educational Development.

^a^ Other did not specify race or ethnicity.

Of all the participants, 3.2% (4 of 126) in the intervention arm and 4.0% (5 of 124) in the control arm reported mild adverse events. There was a total of 15 mild adverse events reported by participants, but none were related to the intervention. No participants were affected by moderate or severe adverse events (eTable 4 in Supplement 2).

### Primary Outcomes

#### Hot Flash Score

On average, hot flash scores decreased for women in both groups, although the overall decrease in hot flash scores was greater for women in the hypnosis group. The mean (SD) hot flash scores for the hypnosis group were 88.7 (61.3) (95% CI, 81.8-95.5) at baseline, 41.3 (50.8) (95% CI, 34.5-48.2) at week 6, and 34.7 (34.4) (95% CI, 27.9-41.6) at follow-up. The mean (SD) hot flash scores for the sham hypnosis group were 94.6 (81.6) (95% CI, 87.7-101.5) at baseline, 55.9 (50.9) (95% CI, 49.0-62.8) at week 6, and 52.8 (49.4) (95% CI, 45.9-59.7) at follow-up. Note that the standard errors and critical values for the 95% CIs were taken from the omnibus ANOVA test.

**Figure 2.**
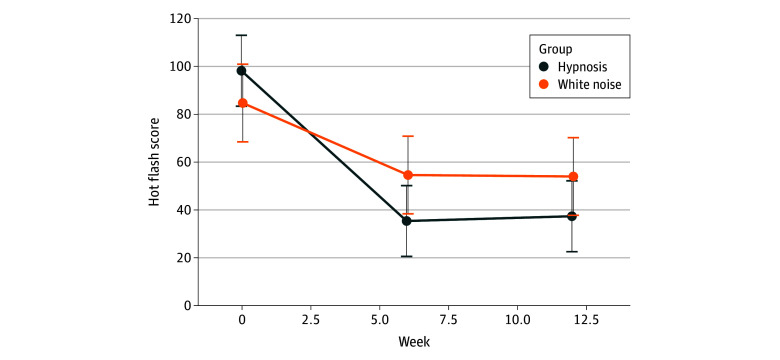
Profile Plots of Hot Flash Outcomes by Treatment Group (Self-Hypnosis vs Sham Control) In the total participant subsample, the hot flash scores for both the self-hypnosis and sham hypnosis groups changed from baseline to week 12, but the self-hypnosis group saw a greater decrease. Error bars indicate 95% CIs for the mean values at each time point during the study.

At 6 weeks, the change in hot flash scores for women in the hypnosis group was 47.4, a 53.4% reduction, while the change in hot flash scores for women in the sham hypnosis group was 38.7, a 40.9% reduction. At the 12-week follow-up, the change in hot flash scores for women in the hypnosis group was 54.0, a 60.9% reduction, while the change in hot flash scores for women in the sham hypnosis group was 41.8, a 44.2% reduction. The Cohen *d* estimates for the decrease in each condition were 0.95 in the hypnosis group and 0.55 in the white noise group from baseline to 12-week follow-up. The profile plot for this comparison is shown in Figure 2. For profile plots of the change in hot flash frequency by hot flash severity, see the eFigure in Supplement 2. The descriptive statistics pooled from the imputed datasets were within 1.0 of the statistics from the original data. In this sample, women who self-administered hypnosis experienced a greater reduction in hot flash scores than women in the sham hypnosis condition, but there was insufficient evidence to conclude with 95% confidence that the change trajectories were not parallel (*F*_1.73, 318.52_ = 2.989; *P* = .06, η^2^*_p_* = 0.02). These findings were consistent with the analyses of all imputed datasets. The study was designed to detect a moderately strong differential treatment effect with moderation, so the mixed ANOVA was slightly underpowered. Nevertheless, all women in the study experienced relief, on average, and those who self-administered hypnosis experienced more relief, on average. Between baseline and week 12, 60.4% of women in the hypnosis intervention (64 of 106 who completed) experienced a 50% or greater reduction in hot flash scores compared with 42.2% (35 of 83 who completed) of women in the sham hypnosis condition (π_diff_ = 0.182 [95% CI, 0.04-0.31]; *z* = 2.49; *P* = .006).

#### Severity of Hot Flashes

The mixed-effects ANOVA compared the frequency of hot flashes by severity type between the hypnosis and sham hypnosis groups. On average, the frequency of hot flashes decreased from baseline to week 12 in both groups for all types of hot flashes (eTable 2 in Supplement 2); however, there were larger effects for moderate hot flash frequency. For mild hot flashes, the hypnosis group showed a mean (SD) difference of 6.3 (14.7) (95% CI, 5.2-9.4) and the white noise group showed a mean (SD) difference of 6.1 (16.3) (95% CI, 4.4-8.8), and the effect was not statistically significant (*F*_1.78, 329.37_ = 0.54; *P* = .42; η^2^*_p_* = 0.003). For moderate hot flashes, at baseline the hypnosis group reported a mean (SD) of 21.6 (21.3) and the white noise group reported a mean (SD) of 20.9 (19.8), but at week 6, the hypnosis group reported a mean (SD) of 8.0 (10.3), and the sham hypnosis group reported a mean (SD) of 12.5 (16.0). At week 12, the hypnosis group reported a mean (SD) of 7.8 (12.0) hot flashes, and the sham hypnosis group reported no further mean decrease. The total mean differences in moderate hot flashes (hypnosis group, 13.8 [95% CI, 10.9-15.9]; white noise group, 8.2 [95% CI, 5.7-10.8]) reflect a small but statistically significant treatment effect (*F*_1.49, 275.80_ = 3.45; *P* = .047; η^2^*_p_* = 0.018). For severe hot flashes, although the mixed-effects ANOVA models did not show statistically significant differences in hot flash trajectories between groups (*F*_1.77, 329.92_ = 2.82; *P* = .07; η^2^*_p_* = 0.015), the women in the hypnosis group reported a mean (SD) of 1.3 (3.7) severe hot flashes at week 12 compared with 6.1 (9.9) at baseline (difference, 4.7 [95% CI, 3.4-6.0]). The sham hypnosis group had a mean of 6.8 severe hot flashes at baseline and 3.8 severe hot flashes at week 12 (difference, 3.0 [95% CI, 0.7-3.4]). For very severe hot flashes, both groups showed a mean reduction in frequency, but very severe hot flashes were rare (hypnosis group: difference, 1.4 [95% CI, 0.0-2.2]; white noise group: difference, 2.5 [95% CI, 0.6-2.8]) and the frequency trajectories were not significantly different (*F*_1.41, 260.73_ = 0.66; *P* = .47; η^2^*_p_* = 0.004).

### Analysis of Patients With History of Breast Cancer

**Figure 3.**
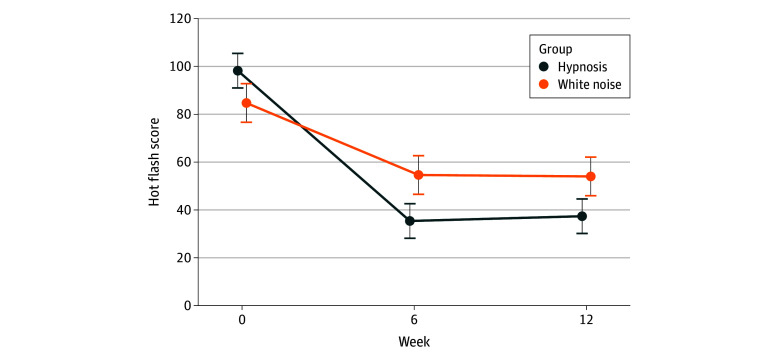
Profile Plots of Hot Flash Outcomes by Treatment Group (Self-Hypnosis vs Sham Hypnosis) for Subgroup of Patients With a History of Breast Cancer In the subsample of patients with a history of breast cancer, the hot flash scores at baseline, week 6, and week 12 are significantly different between the self-hypnosis and sham hypnosis groups. Error bars indicate 95% CIs for the mean values at each time point during the study.

The hot flash score trajectories followed the same patterns within the subsample of patients with a history of breast cancer between the hypnosis and sham hypnosis groups, but the treatment effect was stronger within this subsample. The mean (SD) hot flash score for patients with a history of breast cancer in the hypnosis group was 98.2 (71.6) (95% CI, 91.0-105.4) at baseline, 35.4 (29.0) (95% CI, 28.2-42.6) at week 6, and 37.4 (32.0) (95% CI, 30.1-44.6) at week 12. The mean (SD) hot flash score for patients with a history of breast cancer in the sham hypnosis group was 84.7 (63.9) (95% CI, 76.7-92.8) at baseline, 54.6 (49.7) (95% CI, 46.5-62.7) at week 6, and 54.0 (40.4) (95% CI, 45.9-62.1) at week 12. These differences reflect a larger, statistically significant treatment effect (*F*_1.57, 65.8_ = 7.95; *P* = .002; η^2^*_p_* = 0.16). The profile plot for this comparison is shown in Figure 3.

### Self-Reported Adherence

The self-reported adherence scores were computed as the mean number of reported times in each week each participant practiced her assigned intervention. The mean (SD) adherence score for women with self-hypnosis audio recordings increased from 7.6 (4.4) (95% CI, 7.0-8.3) during week 1 to 10.9 (6.6) (95% CI, 9.6-12.2) during week 6 and then decreased to 8.3 (7.2) (95% CI, 6.8-9.7) at follow-up (during week 12). The mean (SD) adherence scores for those assigned to sham hypnosis decreased from 7.6 (3.6) (95% CI, 7.0-8.3) during week 1 to 7.4 (3.1) (95% CI, 6.8-8.0) during week 6 and decreased again to 5.6 (5.1) (95% CI, 4.5-6.7) at follow-up (during week 12). The mean adherence score was entered to examine the interaction between adherence and the treatment effect within the mixed-effects ANOVA. The interaction term was statistically significant (*F*_1.74, 322.33_ = 10.80; *P* < .001; η^2^*_p_* = 0.06). For women in the hypnosis group, the correlation between mean adherence and each of the hot flash scores was *r* = 0.17 (95% CI, −0.02 to 0.35) for baseline, *r* = 0.26 (95% CI, 0.08-0.43) for week 6, and *r* = 0.11 (95% CI, −0.09 to 0.30) for week 12. For women in the sham hypnosis group, the correlation between mean adherence and each of the hot flash scores was *r* = 0.43 (95% CI, 0.25-0.57) for baseline, *r* = 0.06 (95% CI, −0.14 to 0.26) for week 6, and *r* = −0.04 (95% CI, −0.24 to 0.17) for week 12.

### Hot Flash Related Daily Inference Scale

The HFRDIS secondary outcome was evaluated by item as well as total score. Overall, HFRDIS total scores decreased across the study for the hypnosis and sham hypnosis groups, but the mean reduction was greater for the women in the hypnosis group than those in the sham hypnosis group (27.9-point reduction vs 23.2-point reduction). The mean (SD) HFRDIS scores or the hypnosis group were 49.3 (22.6) (95% CI, 34.5-64.1) at baseline, 25.0 (22.4) (95% CI, 10.1-39.8) at week 6, and 21.4 (20.2) (95% CI, 6.6-36.2) at week 12. The mean (SD) HFRDIS scores for the sham hypnosis group were 47.3 (22.4) (95% CI, 31.1-63.6) at baseline, 29.6 (22.0) (95% CI, 13.3-45.8) at week 6, and 24.2 (20.3) (95% CI, 7.9-40.4) at week 12. The mean (SD) difference of 23.1 (21.5) in the sham hypnosis group represents a 48.8% reduction, and the mean (SD) difference of 27.9 (21.6) in the hypnosis group represents a 56.6% reduction from baseline to week 12; these differences represent a small but statistically significant effect (*F*_1.81, 336.67_ = 3.49; *P* = .04, η^2^*_p_* = 0.02). Similarly, the item-level improvements, based on percentage reduction, was greater for 10 items on the HFRDIS scale. The greatest improvement in the hypnosis group was a 62.6% reduction (mean [SD] difference, 3.1 [2.6]) in hot flash–related daily inference in leisure activities compared with a 51.3% reduction in the sham hypnosis group (mean [SD] difference, 2.3 [2.6]). The item-level changes across time are shown in Table 2.

**Table 2.** HFRDIS Percentage Change by Item by Group

HFRDIS item	% Reduction (% change)^a^			
	Baseline to week 6		Baseline to week 12	
	Hypnosis	White noise	Hypnosis	White noise
Work	46.3 (from 5.0 to 2.7)^b^	29.4 (from 4.5 to 3.2)	56.9 (from 5.0 to 2.2)	46.8 (from 4.5 to 2.4)
Social activities	51.6 (from 4.7 to 2.3)	42.2 (from 4.4 to 2.5)	61.7 (from 4.7 to 1.8)	48.0 (from 4.4 to 2.3)
Leisure activities	55.8 (from 5.0 to 2.2)^b^	38.3 (from 4.6 to 2.8)	62.6 (from 5.0 to 1.9)	51.3 (from 4.6 to 2.2)
Sleep	44.4 (from 7.2 to 4.0)	36.0 (from 7.6 to 4.9)	48.0 (from 7.2 to 3.7)	43.8 (from 7.6 to 4.3)
Mood	48.3 (from 4.9 to 2.5)^b^	35.8 (from 4.7 to 3.0)	54.2 (from 4.9 to 2.2)	49.7 (from 4.7 to 2.4)
Concentration	52.1 (from 5.2 to 2.5)	43.5 (from 4.7 to 2.6)	58.8 (from 5.2 to 2.1)	55.6 (from 4.7 to 2.1)
Relationships with others	52.1 (from 3.6 to 1.7)	43.8 (from 3.7 to 2.1)	59.1 (from 3.6 to 1.5)	53.2 (from 3.7 to 1.7)
Sexuality	49.3 (from 4.4 to 2.2)	43.1 (from 4.4 to 2.5)	58.7 (from 4.4 to 1.8)	48.9 (from 4.4 to 2.3)
Enjoyment of Life	47.9 (from 4.5 to 2.4)^b^	37.3 (from 4.2 to 2.6)	54.1 (from 4.5 to 2.1)	47.3 (from 4.2 to 2.2)
Overall, quality of life	48.8 (from 4.9 to 2.5)	35.1 (from 4.6 to 3.0)	56.3 (from 4.9 to 2.2)	50.9 (from 4.6 to 2.3)

Abbreviation: HFRDIS, Hot Flash Related Daily Interference Scale.

^a^ Percentage change was calculated based on the mean difference divided by the item mean value at baseline. Responses ranged from 0 to 10 and indicated how much hot flashes have interfered with each aspect of life in the past week, where 0 = not at all and 10 = very much so. All reductions from baseline are statistically significant (*P* < .001) within the group.

^b^ *P* < .05 for reduction in daily interference greater for hypnosis group than white noise.

### Perceived Benefit

The association between intervention type and perceived benefit on hot flashes was moderately to relatively strong (χ^2^_5_ = 28.2; *V* = 0.38; *P* < .001). The percentages of participants who reported that their hot flashes improved (ie, “a little better,” “moderately better,” or “very much better”) were 90.3% for the hypnosis group (93 of 103) and 64.3% for the sham hypnosis group (63 of 98) (eTable 3 in Supplement 2). A greater percentage of participants in the hypnosis group than the white noise group reported that their hot flashes got “very much better” (32.0% [33 of 103] vs 18.4% [18 of 98]).

## Discussion

Self-administered clinical hypnosis was shown to be an effective, clinically significant intervention for the treatment of hot flashes due to its efficacy in reducing hot flash scores (ie, frequency and severity) by more than half and yielding improvements in participants’ perception of their quality of life. As reported in prior literature, interventions must achieve a 50% or more reduction in hot flash frequency and daily interference to be considered clinically significant.^37,39,40^ This study’s findings for self-administered clinical hypnosis are consistent with prior randomized clinical trials comparing a therapist-delivered clinical hypnosis intervention with a waiting list^29^ and structured-attention control groups.^28^ Compared with the other most commonly used behavioral intervention, cognitive behavioral therapy, clinical hypnosis continues to consistently demonstrate clinically significant reductions in hot flash symptoms, while findings on cognitive behavioral therapy are more mixed; most studies report improvements in daily interference, perceived bother, or severity but not hot flash frequency reduction.^42^

This study was innovative in its use of an active control group and a self-delivered method of administration. The use of an active control is relatively novel to behavioral intervention studies.^41^ With the use of an active control, participants in both arms experienced some relief from hot flashes, with the white noise control group reporting a 36% decrease in the frequency of severe hot flashes due to the placebo effect. However, those that received hypnosis experienced faster, greater reductions in the severity and frequency of hot flashes compared with the reductions in the control arm.

### Limitations

This study has some limitations, including that most participants were White, non-Hispanic, and had at least 4 years of higher education. This affects the generalizability of the results and warrants further research with a more diverse study population.^43^

## Conclusions

In this randomized clinical trial, the clinical hypnosis group experienced significantly greater reductions of hot flash scores and daily interference from hot flashes compared with the active control condition at week 6. These findings demonstrate that remote delivery of a hypnosis intervention for hot flashes is a safe and effective option for women experiencing hot flashes. Unanswered questions remain regarding the long-term effects of hypnosis on hot flashes, particularly maintenance dosing for continued hot flash prevention.

## Notes

Supplement 1.
Trial Protocol and Statistical Analysis Plan

Supplement 2. **eTable 1.** Schedule of Study Activities **eTable 2.** Descriptive Statistics for Hot Flash Severity Frequency by Treatment Group **eTable 3.** Frequency Distributions (Percentages) of Reported Perceived Benefit of Intervention Across Groups **eTable 4.** Adverse Events **eFigure.** Profile Plots of Hot Flash Outcomes by Treatment Group (Self-Hypnosis vs. Sham Control) and Hot Flash Severity

Supplement 3.
Data Sharing Statement
