# Supplementary material for: Self-Administered Hypnosis vs Sham Hypnosis for Hot Flashes: A Randomized Clinical Trial
Source: JAMA Netw Open. 2025 Nov 11;8(11):e2542537. doi: 10.1001/jamanetworkopen.2025.42537 (PMC12606380; doi:10.1001/jamanetworkopen.2025.42537)
Supplement: Supplement 1. — Trial Protocol and Statistical Analysis Plan [file jamanetwopen-e2542537-s001.pdf]

Self-Administered Hypnosis Treatment for the Management of Hot Flashes in Women: A Randomized Clinical Trial

PREFACE

This research study will fully evaluate the efficacy of a self-administered hypnosis intervention for hot flashes to improve the scalability of this effective hot flash treatment.

The primary objective is to fully evaluate the efficacy of the self-administered hypnosis intervention for hot flashes, with the indication that self-administered hypnosis will significantly decrease vasomotor symptoms compared to the self-administered sham white noise hypnosis control.

Secondary objectives are to evaluate the efficacy of the self-administered hypnosis compared to sham hypnosis for hot flash activity interference, sleep disturbance, anxiety, and perception of benefit; and to explore mediators (perceived stress, cortisol or heart rate variability (HRV)) and moderators of hypnotizability and practice adherence on hot flash reduction.

This is a randomized clinical research trial, involving Baylor University, University of Michigan, and University of Nebraska-Lincoln. Recruitment and data collection will be completed at Baylor University and University of Michigan, with Michigan enrolling 116 participants and Baylor enrolling 131 to achieve total enrollment of 247 participants. Dr. Gary Elkins will serve as the contact PI at Baylor University, and Dr. Deb Barton will serve as the site PI at University of Michigan. Dr. Noel Arring will serve as the Co-Investigator at the University of Michigan. Dr. Tierney Lorenz, (University of Nebraska-Lincoln) will serve as Co-Investigator and afford oversight in matters relative to heart rate variability data collection and analyses. Dr. Grant Morgan (Baylor University) will serve as Co-Investigator and statistician.

Dr. Elkins, PhD, ABPP is a Professor of Psychology and Neuroscience, Director of the Baylor University Mind-Body Medicine Research Laboratory, Director, Baylor University Clinical Psychology Doctoral Program, and is a recognized expert in hypnosis research.

Dr. Barton, RN, PhD., FAAN is a Professor at the University of Michigan School of Nursing, Mary Lou Willard French Endowed Chair of Oncology Nursing, Co-Chair of the Symptom/Quality of Life Steering Committee (NIH) and the Fatigue Study Group for the Multinational Association for the Supportive Care in Cancer, and Co-Leader of the Symptom Management Working Group for NRG Oncology. She has extensive experience in developing and implementing hot flash research and clinical trials to improve symptomatology in oncology.

Dr. Noel Arring, RN, PhD, DNP is an Assistant Professor at the University of Michigan School of Nursing. She has experience with clinical trials for oncology symptom management, particularly with fatigue and the use of complementary therapies.

Dr. Lorenz, PhD is an Assistant Professor in the Department of Psychology and Center for Brain, Biology, and Behavior at the University of Nebraska-Lincoln. She has made multiple contributions to research on the use of HRV as an index of autonomic function in women and has overseen NIH-funded research which employed mobile HRV recording procedures similar to the one in this trial.

Dr. Morgan, PhD is an Assistant Professor at Baylor University. He is a formally trained and experienced research and statistical methodologist and has designed, conducted, and disseminated approximately 70 research or evaluation studies, and is committed to transdisciplinary research in many fields.

Self-Administered Hypnosis Treatment for the Management of Hot Flashes in Women: A Randomized Clinical Trial

Principal Investigators:

Gary Elkins, PhD, Contact PI, Baylor University

Deb Barton, RN, PhD, Site PI, University of Michigan

Supported by:

The National Center for Complementary and Integrative Health

1R01AT009384

Manuscript REVISION HISTORY

Version Number: 1.0
Version Date: February 2018
Summary of Revisions Made: Original Version

Version Number: 2.0
Version Date: May 24, 2018

Version Number: 3.0
Version Date: June 3, 2018

Version Number 4.0 amendment 1
Date: September 16, 2018

Version Number 5.0
Date: October 19, 2018

Version Number 5.0 amendment 2
Date: May 22, 2019 No change to protocol

Version Number 6.0 amendment 3
Date: July 23, 2019

Version Number 6.0 amendment 4
Date: November 4, 2019

Version Number 6.0 amendment 5
Date: April 2, 2020

Version Number 6.0 amendment 6
Date: May 4, 2020

Version Number 6.0 amendment 7
Date: March 1, 2021

Version Number 7.0
Date: April 24, 2023

PREFACE **2**

MANUSCRIPT REVISION HISTORY **5**

TABLE OF CONTENTS **6**

STUDY TEAM ROSTER **9**

PARTICIPATING STUDY SITES **9**

PRÉCIS **10**

1. STUDY OBJECTIVES **11**

1.1 Primary Objective 11
Hypothesis 1.1 11

1.2 Secondary Objectives 11
Hypothesis 2.1 11
Hypothesis 2.2 11
Hypothesis 2.3 11
Hypothesis 2.4 11
Hypothesis 2.5 11
Hypothesis 2.6 11

2. BACKGROUND AND RATIONALE **12**

2.1 Background on Condition, Disease, or Other Primary Study Focus 12

2.2 Study Rationale 12

3. STUDY DESIGN **16**

4. SELECTION AND ENROLLMENT OF PARTICIPANTS **19**

4.1 Inclusion Criteria 19

4.2 Exclusion Criteria 20

4.3 Study Enrollment Procedures 20

5. STUDY INTERVENTIONS **21**

5.1 Interventions, Administration, and Duration 21
5.1.1 Self-Administered Hypnosis Intervention 21
5.1.2 Self-Administered Sham White Noise Comparator 21

5.2 Handling of Study Interventions 22

5.3 Concomitant Interventions 22
5.3.1 Allowed Interventions 22
5.3.2 Required Interventions 22

5.3.3 Prohibited Interventions 23

5.4 Adherence Assessment 23

6. STUDY PROCEDURES 23

6.1 Schedule of Evaluations 23

6.2 Description of Evaluations 23

6.2.1 Screening Evaluation 23

6.2.2 Enrollment, Baseline, and/or Randomization 25

6.2.3 Blinding 28

6.2.4 Follow-up Visits 29

6.2.5 Completion/Final Evaluation 29

7. SAFETY ASSESSMENTS 30

7.1 Specification of Safety Parameters 30

7.2 Methods and Timing for Assessing, Recording, and Analyzing Safety Parameters 30

7.3 Adverse Events and Serious Adverse Events 31

7.4 Reporting Procedures 31

7.5 Follow-up for Adverse Events 32

7.6 Safety Monitoring 32

8. INTERVENTION DISCONTINUATION 32

9. STATISTICAL CONSIDERATIONS 33

9.1 Primary Objective 33

Hypothesis 1.1 33

9.2 Secondary Objectives 33

Hypothesis 2.1 33

Hypothesis 2.2 34

Hypothesis 2.3 34

Hypothesis 2.4 34

Hypothesis 2.5 34

Hypothesis 2.6 34

9.3 Sample size and randomization 34

9.4 Definition of Populations 36

9.5 Interim Analyses and Stopping Rules 36

9.6 Outcomes 37

9.6.1 Primary Outcome 37

9.6.2 Secondary Outcomes 37

9.7 Data Analyses 39

10. DATA COLLECTION AND QUALITY ASSURANCE 41

10.1 Data Collection Forms 41

10.2 Data Management 43

10.3 Quality Assurance 44
10.3.1 Training 44
10.3.2 Quality Control Committee 44
10.3.3 Metrics 44
10.3.4 Protocol Deviations 44
10.3.5 Monitoring 44

11. PARTICIPANT RIGHTS AND CONFIDENTIALITY 45
11.1 Institutional Review Board (IRB) Review 45
11.2 Informed Consent Forms 45
11.3 Participant Confidentiality 45
11.4 Study Discontinuation 46

12. COMMITTEES 46

13. PUBLICATION OF RESEARCH FINDINGS 46

14. REFERENCES 47

STUDY TEAM ROSTER

Gary Elkins, PhD
One Bear Place #97243
Waco, TX 76798-7243
254-296-0643
(FAX) 254-296-9393
gary_elkins@baylor.edu

Deb Barton, RN, PhD
400 N. Ingalls, Room 4304
Ann Arbor, MI 48109-1274
734-763-3868
debbartn@med.umich.edu

Tierney Lorenz, PhD
C66 East Stadium
University of Nebraska-Lincoln
Lincoln, NE 68510-0156
Tierney.Lorenz@unl.edu

Grant Morgan, PhD
One Bear Place #97301
Waco, Texas 76798-7301
254-710-7231
grant_morgan@baylor.edu

Catherine Van Poznak, MD
Michigan Cancer Center
cvanpoz@med.umich.edu

Noel Arring, RN, PhD, DNP
400 N. Ingalls, 4356
Ann Arbor, MI 48109-1274
nmarring@med.umich.edu

PARTICIPATING STUDY SITES

Baylor University
Gary Elkins, PhD
Contact/Site PI
One Bear Place #97243
Waco, TX 76798-7243
254-296-0643
(FAX) 254-296-9393
gary_elkins@baylor.edu

University of Michigan
Deb Barton, RN, PhD
Site PI
400 N. Ingalls, Room 4304
Ann Arbor, MI 48109-1274
734-763-3868
debbartn@med.umich.edu

PRÉCIS

Full Study Title

Self-Administered Hypnosis Treatment for the Management of Hot Flashes in Women: A Randomized Clinical Trial

Objective

The long-term goal of this program of research is to determine effective and safe non-hormonal interventions for menopausal symptoms, and the primary objective of this study is to fully evaluate the efficacy of the self-administered hypnosis intervention for hot flashes, with the indication that self-administered hypnosis will significantly decrease vasomotor symptoms compared to the self-administered sham white noise hypnosis control at 6 weeks. Secondary objectives include evaluating the effect of the intervention on hot flash activity interference, sleep disturbance, anxiety and perception of benefit (secondary outcomes). Exploratory outcomes include the role of mediators (perceived stress, cortisol, heart rate variability) and moderators (hypnotizability and practice adherence).

Design and Outcomes

This randomized, two arm clinical trial will evaluate the efficacy of self-administered hypnosis for the treatment of hot flashes in postmenopausal women, 18 years or older with a self-reported history of a minimum of four hot flashes per day or 28 hot flashes per week at baseline.

Participants will maintain a hot flash diary for the first week without using the audio-recorded intervention to establish a baseline data point. This will be followed by six weeks of using the self-management materials (hot flash diaries, hypnosis practice log, booklets, and audio recordings). Participants will complete primary endpoint assessments at the end of Week 6. Participants will also be asked to complete the assessments at the end of Week 12 (Follow-up), to evaluate treatment sustainability as a secondary outcome.

Interventions and Duration

The intervention, self-administered hypnosis with hypnotic induction, will be compared to a self-administered sham white noise hypnosis group (no hypnotic induction), in two equivalently structured groups. Participants in both arms will have two initial visits in person (or by telephone or video conference during COVID-19 restrictions) with the study research contact staff to complete baseline questionnaires, establish rapport for effective follow up calls with respect to adherence and be educated about their assigned treatment. The self- administered sham white noise hypnosis group delivery will be structurally equivalent to include the same research education and phone interaction as the self-administered hypnosis, but the audio-files do not contain hypnotic inductions.

Sample Size and Population

Two hundred thirty-two women, 18 or older who meet the criteria outlined in Sections 4.1 (Inclusion Criteria) and 4.2 (Exclusion Criteria) will be randomized in the study; with approximately half randomized at each study site (i.e., 116 at Baylor University and 116 at University of Michigan).

Further, at each site the 116 participants will be randomized to participate in one of the two groups, self-administered hypnosis or self-administered sham white noise hypnosis, providing for 58 participants per group at each site.

1. STUDY OBJECTIVES

1.1 Primary Objective

The primary objective is to fully evaluate the efficacy of the self-administered hypnosis intervention for hot flashes, with the indication that self-administered hypnosis will significantly decrease vasomotor symptoms compared to the self-administered sham white noise hypnosis control.

Hypothesis 1.1:
Self-administered hypnosis will significantly decrease vasomotor symptoms (hot flash score of frequency and severity) compared to the self-administered sham white noise hypnosis at 6 weeks.

1.2 Secondary Objectives

Secondary objectives are to evaluate the efficacy of the self-administered hypnosis compared to self-administered sham white noise hypnosis for hot flash activity interference, sleep disturbance, anxiety and perception of benefit; with the expectation that there will be significant improvements among participants in the self-administered hypnosis group when compared with the self-administered sham white noise hypnosis group at 6 weeks. Further, we will evaluate the effects of self-administered hypnosis compared to the self-administered sham white noise hypnosis control at 12 weeks, for all secondary outcomes except perception of benefit.

Hypothesis 2.1

Compared to self-administered sham white noise hypnosis control, the self-administered hypnosis intervention will result in significant improvements in hot flash activity interference, sleep disturbance, and anxiety at 6 and 12 weeks, and perception of benefit at 6 weeks.

Additionally, mediators (perceived stress, cortisol, and heart rate variability (HRV) and moderators (hypnotizability and practice adherence) of hot flash reduction will be explored to address the following hypotheses:

Hypothesis 2.2

A decrease in perceived stress will be indicated in the self-administered hypnosis group due to the intervention, which will correspond and contribute to further decrease of the hot flash score at 6 and 12 weeks.

Hypothesis 2.3

Diurnal salivary cortisol will demonstrate better circadian rhythms (steeper slopes) in those with larger hot flash reductions and in the self-administered hypnosis group compared to participants in the self-administered sham white noise hypnosis at 6 weeks.

Hypothesis 2.4

Self-administration of hypnosis and greater hot flash reductions will result in higher resting and 24-hour parasympathetic activity, reflected as higher HRV at 6 weeks.

Hypothesis 2.5

Higher hypnotizability scores and better adherence rates will be associated with greater reductions in hot flash scores in the self-administered hypnosis group.

Hypothesis 2.6

Self-administered hypnosis will significantly decrease vasomotor symptoms (hot flash score of

frequency and severity) compared to the self-administered sham white noise hypnosis at 12 weeks.

2. BACKGROUND AND RATIONALE

2.1 Background on Condition, Disease, or Other Primary Study Focus

Hot flashes are a prevalent symptom after menopause (natural and surgical), but also after treatment for breast cancer. It is estimated that over 25 million women in the United States have hot flashes (HFs),[1, 2] with up to 80% of women in the general population reporting hot flashes during the menopause transition[3] and lasting an average of 7.4 years;[4] 96% of women with breast cancer reporting hot flashes soon after beginning anti-cancer therapy[5] and up to 73% reporting hot flashes 6 years after diagnosis, even after endocrine therapy has been completed.[6] Women may, however, experience hot flashes for 15 or more years.[7] Much of the descriptive information about the prevalence, duration and impact of hot flashes has come from older studies,[3] such as the Study of Women's Health Across the Nation (SWAN). Nonetheless, hot flashes are a symptom that impacts a very large number of women, both with and without a history of breast cancer, and they can persist for an extended time.

Hot flashes negatively impact health related quality of life. Over half of the women with a history of breast cancer report their hot flashes as severe.[8, 9] Hot flashes can cause sweating, discomfort, anxiety, fatigue, and can interfere with sleep leading to adverse health outcomes.[10-16] One large population based study of 2,703 post-menopausal women found that hot flashes negatively impact work (46%), sleep (82.0%), mood (68.6%), total energy level (63.3%), and overall quality of life (69.3%).[13] Descriptive studies of women with a history of breast cancer and the general community link hot flashes to poorer emotional and social functioning in addition to increased anxiety and poor sleep.[5, 14, 16] There are data supporting the link between poor sleep and increased mortality as well as risk for chronic diseases.[11, 12] Therefore, effective treatment of hot flashes can have broad positive effects on health and health related quality of life.[13]

There is a pressing need for safe and effective options for treatment of hot flashes for breast cancer survivors. Until 2000, the gold standard treatment of HFs was estrogen-based therapy, which reduced both the frequency and severity of hot flashes by up to 90%.[10, 15] However, research has shown that hormone replacement therapy is associated with several side effects, such as the increased incidence of venous thromboembolism, endometrial cancer, breast cancer, and stroke, as well as incidence of metastatic tumors and death among lung cancer patients.[17-19] A large cohort study confirmed that there was a clinically and statistically significant increased risk of breast cancer reoccurrence in BCS who took hormone replacement therapy.[20] Although current research demonstrates that consideration of factors such as drug formulation, administration route, and age of initiation can help physicians maximize the benefit-to-risk ratio of these agents for individual women,[19] hormone therapies are still associated with a non-negligible risk of untoward side effects and are mostly contra-indicated in women with a history of breast cancer.[19, 21] Concerns about side effects and risks prompted the search for non-hormonal alternatives to treat hot flashes.[22]

2.2 Study Rationale

Alternatives to hormone therapy for the reduction of hot flashes are limited. A common alternative to hormone-based treatment for HFs are antidepressants and the medication gabapentin. However, these effects are believed to be modest compared to that of estrogen therapy.[23] Meta-analyses and pooled analyses demonstrate a 60% reduction with serotonergic antidepressants and the anti-seizure drug gabapentin.[24-26]

Gabapentin must be taken three times per day for optimal results, making it inconvenient.[24] Undesirable side effects and stigma can limit the use of antidepressant medication as a treatment for hot flashes. Side effects of serotonergic antidepressants include dry mouth, headaches, nausea, drowsiness, increased anxiety, and sexual dysfunction,[27] while gabapentin is associated with dizziness, sedation, fluid retention, and weight gain.[28] To summarize, non-hormonal treatments for HFs are 20 to 30% less effective than estrogen based treatment and are fraught with the potential for significant side effects.

In an attempt to address the limitations of pharmacologic interventions, in the last decade behavioral interventions such as paced respiration, cognitive-behavioral therapy, yoga, and mindfulness therapy have been investigated as treatments for HFs.[29-31] Behavioral treatments are important, as in at least one study, women reported a preference for behavioral over medical treatments for hot flashes.[32] While studied behavioral treatments have indeed demonstrated few unwanted side effects and a reduction in emotional distress, they have not been shown to actually reduce hot flashes to a clinically significant degree,[33-36] clinical significance defined as 50% or greater reduction in frequency of hot flashes.[37] Therefore, more effective behavioral options are needed.

Hypnosis is an effective option for hot flashes. Dr. Elkins and colleagues have developed a hypnosis intervention involving standardized audio recordings of hypnosis for home practice and individualized hypnosis inductions delivered by a clinical researcher. This treatment reduced the frequency and severity of Hot Flash Scores (HFS) by approximately 80% *on average* in postmenopausal women with and without a history of breast cancer, which is comparable in efficacy to that of hormone replacement therapy.[38, 39] The positive effect of hypnosis on hot flashes has been replicated by Dr. Barton and others.[40, 41] This burgeoning data has resulted in hypnosis being added to the clinical practice guidelines of the North American Menopause Society[42] as having level 1 evidence. The current limitation for this is scalability. There are few practitioners in the US who provide hypnosis as a therapeutic strategy and the locations of these practitioners are extremely limited. Hence, there is a critical need to develop and provide evidence for the effect of a hypnosis intervention that can be self-administered by women. This effort is in alignment with a recent Institute of Medicine (now National Academy of Medicine) report on women's health, highlighting the need to move research from clinical guidelines to widespread practice.[43]

This critical next step, evaluating the efficacy of a fully self-administered hypnotic intervention, is needed to make this intervention broadly available to women. Since clinician directed hypnosis has been shown to be efficacious, our scientific premise is that self-administered hypnosis will be an effective, accessible treatment without side effects.

The physiology of hot flashes is not definitively known due to limited animal models of natural menopause.[44, 45] In addition, the mechanisms through which hypnosis can decrease hot flashes are not known, and the models which exist rarely incorporate interactions between somatic and psychological factors. Nevertheless, experimental research suggests that dysregulation of the autonomic nervous system (ANS) may contribute to hot flash symptomatology, both in terms of objective rises in temperature, and in subjective discomfort. Researchers for this study hypothesize that self-administered hypnosis will have two key effects on hot flash symptoms. First, suggestions for cooling with images integrating cool breezes, cool water, snow, and air-conditioned air will promote a subjective sense of comfort during a hot flash. Several studies have found that increased sympathetic nervous system (SNS) activity heightens subjective discomfort in response to noxious temperature stimuli[46-49] and that increased parasympathetic nervous system (PNS) activity significantly increases the threshold at which heat is perceived as uncomfortable or intolerable.[50, 51] Cooling hypnosis suggestions may decrease SNS activity during a hot flash[52, 53] which in turn would increase the discomfort threshold. Second, a relaxation response may increase baseline PNS activity, which in turn will decrease the chance of small increases in SNS activity triggering a hot

flash. Sympathetic recruitment during a hot flash has been well documented.[54-56] Importantly, in individuals displaying SNS hyperactivity (e.g., during stress), even small increases of SNS activity may trigger a hot flash.[57, 58] Blockade of SNS hyperactivity has been shown to control hot flashes better than placebo;[59, 60] similarly, behavioral interventions that reduce SNS hyperactivity such as yoga and acupuncture can also reduce number and severity of hot flashes.[61, 62] Several studies show that hypnosis results in a decreased sympathetic activity.[52, 63, 64]

Heart rate variability (HRV) has been established as a biomarker of autonomic balance with well validated clinical and research guidelines.[65, 66] Cortisol is a widely accepted biomarker for hypothalamic-pituitary-adrenal axis (HPA) recruitment in response to stress with diurnal rhythms being particularly well validated.[67, 68] This study will evaluate HRV and cortisol to examine physiologic mechanisms by which hypnosis influences hot flashes.

The purpose of the present research study therefore is threefold: 1) to test the efficacy of a self-administered hypnosis intervention approach versus a self-administered sham white noise hypnosis arm, to reduce hot flash scores (severity and frequency) in women with and without a history of breast cancer; 2) to evaluate the effect on related menopausal symptoms of sleep disturbance, anxiety and daily interference of hot flashes, and 3) to assess the underlying mechanisms of the effects of the self-administered hypnosis on hot flashes.

Self-administered hypnosis is an innovative delivery platform for an effective intervention for the treatment of hot flashes. This study is innovative because it seeks to evaluate an intervention that is typically delivered by trained therapists, which limits its access. In this study a structured self-administration procedure that preserves important aspects of the original intervention, namely hypnotic induction with suggestions of relaxation and coolness, will be administered. The recorded hypnotic inductions for this study begin with a focus of attention, then continue with suggestions for relaxation, deepening, and mental imagery and suggestions for experiencing coolness and reducing hot flashes (69, 70) An example of the hypnotic induction and transcript is shown in Supplements/Appendices 15.18 .The data on therapist-delivered hypnosis for hot flashes is compelling to the point of being added to clinical guidelines,[42] but the scalability of this intervention is limited by trained therapists and reimbursement providing a barrier in the realm of access to care. Therefore, there is a fundamental need for a less personnel-intensive and easily accessible version of this treatment. Although home practice using audio recordings has been found to achieve prolonged beneficial effects in the hypnotherapy of chronic health conditions[70], a completely self-delivered approach has not yet been evaluated. If this intervention is effective, it would allow women to utilize hypnosis without a hypnotherapist, providing opportunities for broad dissemination. In addition, if this study demonstrates hypnosis has efficacy delivered in this way, relief for other symptoms could be evaluated using self-administered hypnotic interventions tailored to those symptoms.

This is the first study to evaluate potential mechanisms of effects of hypnosis for hot flashes. Recent findings suggest that anxiety and stress can potentiate hot flashes, and that autonomic imbalance may be an underlying etiology.[54, 55, 57, 71] This study will assess the role that autonomic activity and perceived stress plays in hypnosis' effects on hot flashes using stress biomarkers and heart rate variability along with self-report measures**.** It is difficult and expensive to measure transmitter activity in the brain, as peripheral concentrations have not been validated to represent central concentrations adequately.[72] Using heart rate variability to be able to measure the amount of sympathetic versus parasympathetic activity at baseline and evaluate the potential shift in the ratio of those two is innovative and will provide information not yet obtained related to hypnosis and hot flashes. We can then compare this shift in ratio to diurnal cortisol and self-report measures, and to self-reported stress and anxiety. Therefore, we will be able to evaluate cortisol and HRV as potential biomarkers and understand autonomic balance and stress reduction as mechanisms through which hot flashes are reduced with the use of hypnosis. We feel that these findings will provide influential

information as we move toward advancing our understanding and knowledge of the mechanisms of hypnosis.

Preliminary data for the evaluation of a completely self-administered hypnosis intervention has been completed and published by Dr. Elkins' research team.[73] In 13 women with a mean age of 55 years, hot flash frequency was reduced an average of 72% over 5 weeks, with 11/13 women experiencing over 60% reduction. At each of the 5 weeks, participants listened to an audio recording on their own and did not interact with a hypnotherapist. A graph illustrating the hot flash score reduction (frequency X severity) is shown in **Figure 1**, demonstrating a 76% reduction, consistent with provider delivered effects.

Two NIH/NCCIH funded randomized trials completed and published by Dr. Elkins' group demonstrate the effectiveness of therapist-conducted hypnosis, one in 187 post-menopausal women in the general population[39] and one with 60 women with a history of breast cancer.[38] The earlier study in women with breast cancer utilized a wait list control group. The general (post-menopausal) population study employed an attention control group. Primary outcomes were frequency of hot flashes taken from daily diaries and hot flash score (calculated from hot flash frequency and severity ratings).

Figure 1. *Weekly reductions in hot-flash score*

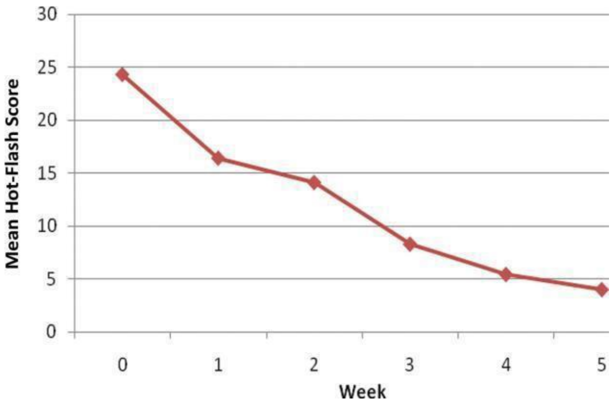

Secondary outcomes included measures of hot-flash related daily interference (Hot Flash Related Daily Interference Scale [HFDIS]), sleep quality (Pittsburgh Sleep Quality Index [PSQI]), anxiety (HADS Anxiety subscale [HADS-A]), and depression (Center for Epidemiologic Studies Depression Scale [CES-D]). Self-reported frequency and hot flash score were assessed by daily diaries, while secondary measures were assessed with questionnaires at weeks 0 (baseline), week 6, and week 12. Mean reductions in hot flash score, (representing both frequency and severity) were 16.72 in the Hypnosis Group (71.36%) at week 6 from baseline, as compared to a 1.91 reduction in hot flash score (8.32% reduction) in control, (p<.001, 95% CI 38.84-47.85). At week 12 follow-up, participants in the hypnosis intervention reported a continuing reduction of 18.83, (80.32%) from baseline as compared to a 3.53 (15.38%) in the control condition. Mean differences in hot flash scores at week 12 follow-up were significant between conditions (p<.001, 95% CI 12.60-17.54.[39]

Reductions in subjective mean hot flash frequency from baseline to week 6 in the hypnosis intervention showed a mean reduction of 48.07 hot flashes (63.87%) from baseline as compared to a 6.95 reduction in control (9.24%). The mean difference in hot flash frequency between conditions was significant, (p<.001, 95% CI 38.84-47.85). Mean reductions continued into week 12, with follow-up reports of hot flash frequency showing a mean reduction of 55.82 hot flashes from baseline (74.16%), as compared to a 12.89 hot flash reduction (17.13%) for participants in the structured attention condition (p<.001, 95% CI 36.15-49.67). Hot flash related interference in daily activities, sleep quality, anxiety and depression were also all significantly reduced from baseline to week 6 for those receiving hypnosis over the structured attention control group.[39]

The study completed with 60 breast cancer survivors,[38] used a "no treatment" control. Hot flash score was reduced 68% in the hypnosis arm and was significantly better than the control group (p<.001), as were reductions in anxiety and hot flash interference per the HFRDIS.

Research also conducted through a multiple PI grant with Dr. Elkins and Dr. Barton supported by NCCIH and NCI (NCT 01000623) in a mixed population of women with and without a history of breast cancer evaluated whether a combination of venlafaxine and hypnosis would provide a greater hot flash reduction than either therapy alone, but also compared the combination to a double placebo arm. A placebo hypnosis condition, called "focused attention" was developed using white noise and this arm combined a placebo pill (matching the venlafaxine) with the white noise placebo hypnosis condition.[41] The double placebo arm demonstrated placebo effects 25% consistent with what has been seen in a large body of previous hot flash research[24, 37, 74] and was successfully used to engage participants in this 4 arm RCT. **Figure 2** demonstrates the results of this study, showing that the hypnosis alone arm was equal to the venlafaxine alone arm, however, only the hypnosis arm was significantly better than placebo. In addition, the combination of venlafaxine and hypnosis was NOT more effective in reducing hot flashes than either treatment alone. The hypnosis reduction of 50% was less than that seen in Dr. Elkins' study as the study was done with Dr. Barton's team that included RN's with varied educational backgrounds (bachelor's, master's, PhD), but all new to this intervention (i.e., hypnosis). Although this pilot study clearly proved licensed providers can be trained to effectively deliver hypnosis, there was a learning curve with respect to hypnosis delivery that explains the lower mean reductions (50% versus 70%).

Figure 2. *Average Hot Flash Scores by Week by Group*

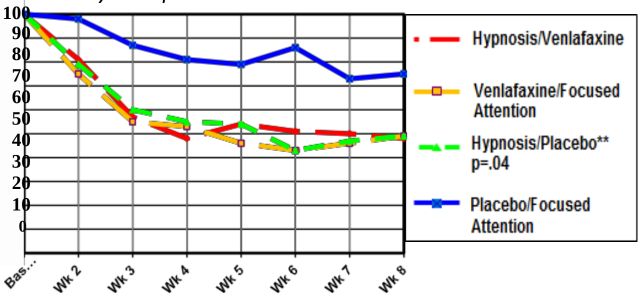

This self-administered protocol is designed to bypass this issue of providers gaining competency and be sufficiently effective in reducing hot flashes and improving associated symptoms of sleep and anxiety. This study also demonstrates the appropriateness of the "sham hypnosis" as a control arm as the focused attention was actually sham hypnosis using white noise.

Yet another group has recently published results of a small pilot study demonstrating that hypnosis can improve hot flashes significantly more than gabapentin.[40] The gabapentin group reported hot flash reductions of 33% while the hypnosis group reported hot flash reductions of 80%.

Therefore, three different investigative teams have been able to demonstrate significant reductions in hot flashes that have been better than or equal to known effective pharmacologic therapy or control groups structured to control for non-specific effects. Additionally, Dr. Barton has been part of the research team that developed the methodology that has been used in numerous hot flash studies across the country, specifically, the development and utilization of the hot flash diary and the primary outcome of the hot flash score.[37, 75] Therefore, this research study is the next logical step to evaluate a more scalable hypnosis intervention that would involve self-administration and allow for this effective intervention to be broadly accessible. A co-investigator with expertise in heart rate variability (HRV) has been added to this research team. Dr. Tierney Lorenz has completed studies incorporating HRV and has published several technical papers on the use of HRV as an index of autonomic function in women[76, 77] as well as papers using HRV in to assess autonomic mechanisms of psychological treatments for mental and physical health sequelae of childhood sexual abuse.[78, 79]

3. STUDY DESIGN

This study is designed as a randomized, two arm clinical trial to evaluate the efficacy of self-administered hypnosis for the treatment of hot flashes. The primary outcome will be the hot flash score,

which is determined from the frequency and severity of hot flashes obtained using the Hot Flash Daily Diary and will be determined at the 6-week data point.

Secondary endpoints will include: daily activity interference and overall quality of life measured by the Hot Flash Related Daily Interference Scale (HFRDIS), sleep disturbance measured by the Pittsburgh Sleep Quality Index (PSQI), and anxiety assessed by the PROMIS Emotional Distress - Anxiety Scale the Hot Flash Daily Diary at 6 and 12 weeks, and the Subject Global Impression of Change, at 6 weeks only.

It is expected that the hypnosis intervention will lead to decreased sympathetic nervous system (SNS) activity and increased parasympathetic tone. This shift in autonomic balance will have beneficial effects on hot flash score (frequency and severity-the primary outcome). Improvement in hot flashes will result in improvement in the secondary outcomes (activity interference, quality of life, sleep quality, and anxiety). Additionally, it is expected that perceived stress will decrease in the treatment group due to the intervention, which will correspond to further decrease of the hot flash score, but act as a mediator; and this will be measured with the Perceived Stress Scale. Because both autonomic arousal[71] and psychological stress[57] are believed to play a critical role in the initiation of hot flashes, we hypothesize that improvement in primary and secondary outcomes will be mediated by perceived stress and the autonomic response to hypnosis (**Figure 1**).

Figure 1: *Conceptual Framework*

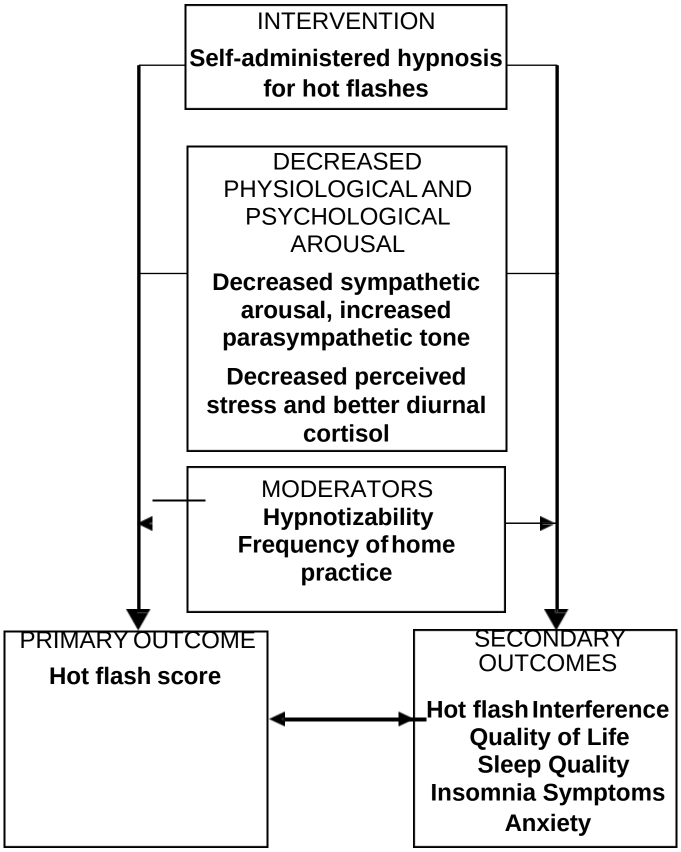

The comparison condition, called sham white noise hypnosis, is structured to account for non-specific effects of general time and attention, in addition to non-hypnotic audio-recordings to evaluate whether this self-administered hypnosis will reduce hot flashes more than an appropriate control group. This represents a rigorous RCT design.

There will be 232 participants who are post-menopausal or breast cancer survivors and wish to avoid hormonal therapy to treat hot flashes.

Eligible participants will be randomized to either self-administered hypnosis intervention or self-administered sham white noise hypnosis which includes listening to white noise. Randomization will be accomplished by the statistical team using stratified permuted block randomization with fixed blocks of size four. Two strata will be used: site (University of Michigan or Baylor University) and participant subgroup (post-menopausal or breast cancer). All randomization block permutations will be generated, and one permutation will be randomly assigned to each block. The randomization information will be printed on individual cards, each of which will be placed in a brown security envelope and sealed (one card per envelope). The envelopes will be chronologically numbered and assigned according to that order. It is expected that each site (Baylor University and University of MI) will each recruit approximately half of the participants, but each site will receive two sets of 116 envelopes – one set of 116 for each participant subgroup. It is not expected that there be an equal distribution of participants who are post-menopausal or breast cancer survivors; rather, the randomization strategy ensures an equal distribution of each subgroup into the self-administered hypnosis intervention or self-administered sham white noise hypnosis condition regardless of the proportion of each participant subgroup within site. For example, if Baylor University randomizes 100 participants who are post-menopausal and 16 who are breast cancer survivors, having two sets of 116 randomization envelopes ensures that (1) there are enough envelopes and (2) there are 50 post-

menopausal and 8 breast cancer survivors in each condition. Therefore, one set of sealed envelopes for post-menopausal participants with group assignment and one set of sealed envelopes for breast cancer survivors with group assignment will be provided to both Dr. Elkins' team and Dr. Barton's team. The randomization list will be kept by the statistician and person(s) who will randomize participants, educating them about their treatment assignment, and not accessible to persons who will be educating, obtaining consent from the participants, collecting data, or interacting in any other capacity which involves contact with participants, as this may possibly cause bias in the recruitment process. The unused randomization envelopes will be destroyed.

The accrual and enrollment duration for the entire trial is expected to be approximately 39 months. (**Table 1**) Individual participant time in the study will be approximately 13 weeks (i.e., baseline week through week 12 follow-up).

Table 1

**MONTHS 1-9**<br>**Training; preparation; recruitment**	**MONTHS 10-42**<br>**Recruitment; sessions; data collection;** data entry	**MONTHS 43-47**<br>**Complete Data Collection**	**MONTHS 48-60**<br>**Data Management; Analyses; Write- ups**
Train staff, research coordinator, and research contact staff	Recruit participants	Complete study visits	Finalize data cleaning
Develop and assemble study materials, record all audio recordings	Format data files, prepare data sets, enter data	Collect Follow-Up data and complete data entry	Analyze data
Regulatory procedures to begin recruitment		Begin planning manuscripts and presentations	Prepare manuscripts and present papers/posters

Eligible participants will be randomly assigned to either the self-administered hypnosis intervention group or the self-administered sham white noise group. The study hypothesis will be blinded such that all participants will be told there are two different self-administered hypnosis programs being evaluated. Both groups will meet with a research contact staff two times (consent/baseline visit and randomization/education visit) in person (or by telephone or video conference during COVID-19 restrictions) and further contact will be by phone, with approximately 10-day intervals between calls for a total five phone calls over a 6-week span (**Table 2)**. One final phone call will occur at week 11 to remind participants about the 12 week data collection. The baseline appointment will include completion of all baseline questionnaires (HFRDIS, PSQI, PROMIS Emotional Distress-Anxiety, and PSS). Participants will also be provided with a hot flash daily diary (for 7 days of baseline data collection), saliva collection materials, and a

Table 2: *Weekly participant contact*

**Baseline** In-person (or telephone or video conference during COVID-19 restrictions) Research Staff Interaction followed by *7-day Hot Flash Daily Diary; then* ***Randomization/ education*** *visit*	*During the week of* ***Week 1***<br>**Call 1**	**Call 2**	**Call 3**	**Call 4**	**Call 5**
	Research Staff Phone contact	Research Staff Phone contact	Research Staff Phone contact	Research Staff Phone contact	Research Staff Phone contact

Polar V800 HRV device (each for two days of baseline sample/data collection) prior to using the recordings. The intent of the baseline visit is to establish rapport with the participant and to ensure understanding of the baseline data collection related to the saliva, heart rate and hot flash diary. Within 30 days of the baseline data collection, the participants will be scheduled for a randomization visit where they will learn in detail about implementing their assigned intervention. Participants in the hypnosis group will also receive the hypnotizability assessment at the randomization visit. The study phone visits are not therapeutic for hot flashes. The phone calls will maintain contact to encourage practice of the self-administered programs and answer study related questions. In addition, administrative phone calls will occur as needed to assure data collection has been sent and received, as well as to answer questions relating to participation in the study. Participants in the self-administered hypnosis group will also receive the hypnotizability assessment (Elkins Hypnotizability Scale [EHS] which is also not a therapeutic intervention. The EHS-CF will be administered if visit is by telephone or video conference (during COVID-19 restrictions).

Audio recordings will be developed and distributed to the participants based on individual randomization. The recordings will be available to the participants as pre-recorded digital audio files, flash drive, or CDs and be given during the randomization visit. The audio recordings will be mailed to the participant if the randomization visit will occur through phone or video conference due to COVID-19 restrictions or participants will be provided with digital audio files. For participants who prefer a flash drive or CDs, those will be provided. Additionally, if a participant prefers to use CDs, but does not have a CD player, one will be provided. Based on their past research experience, Drs. Elkins and Barton will facilitate this process in that, through years of clinical practice and research, it has been found that when women are asked about a place that is safe, positive and relaxing for them, only a few "scenes" of imagery come up: beach, lake, forest, and mountains.[69]

Therefore, as illustrated in **Table 3**, the recordings for the Self-Hypnosis Intervention Program will be created using preferred imagery for the inductions provided for Weeks 1 and 2. For Week 3, a hypnosis induction will be included which will instruct and allow for participants to individualize and focus on a personal imagery that prompt feelings of coolness and comfort. This type of induction addresses the needs of women who would respond better to a less specific suggestion. The Week 4 recording will provide instruction in self-hypnosis without the use of the recordings. Week 5 will guide the participant as they move to integrate the intervention into their daily lives using both methods (recorded and non-recorded self-hypnosis practice); and participants will be encouraged to use the recording of their choice during the final week (Week 6) of the program.

Table 3: *Audio-recording contents*

Week	Self-Hypnosis Intervention	Sham White Noise Hypnosis Control
1	Hypnotic Induction with imagery 1	White Noise
2	Hypnotic Induction with imagery 2	White Noise
3	Hypnotic Induction Individualization	White Noise
4	Hypnotic Induction Self-Hypnosis	White Noise
5	Hypnotic Induction Integration	White Noise
6	Preferred recording(s) from Weeks 1-5	White Noise

The self-administered sham white noise hypnosis recordings will be one recording of white noise, which will time-match the audio recordings designed for the self-hypnosis intervention program. Participants in the self-administered sham white noise hypnosis group will use the hot flash education recording week 1 and the white noise recording weeks 2-6.

Each participant will receive a toolkit in one well organized binder that includes specific instructions for each week, tailored to the treatment assignment, instructions about the saliva collection and HRV measurement, the hot flash diaries, practice logs, and questionnaires for weeks 6 and 12. The toolkit will also include sufficient addressed, stamped envelopes for questionnaire and saliva and HRV return.

4. SELECTION AND ENROLLMENT OF PARTICIPANTS

4.1 Inclusion Criteria

Participants must meet all inclusion criteria to participate in this study.

- Postmenopausal as defined by one of the following criteria:

1) no menstrual period in the past 12 months
2) chemical menopause (LHRH antagonists)
3) surgical menopause (bilateral oophorectomy)
4) evidence of menopause status per FSH and estradiol levels per institutional guidelines.

- Self-reported history of a minimum of 4 hot flashes per day or 28 hot flashes per week at baseline
- Age over 18 years and able to give consent for participation in the study
- Participants on antidepressants for hot flashes are allowed on study if the antidepressant has been for at least 2 months, there are no plans to change the dose or antidepressant, and the number of hot flashes meets the eligibility criteria of at least 4 hot flashes per day or 28 per week.
- Have discontinued other putative therapies for hot flashes for at least one month prior to enrollment (Vitamin E will be allowed)
- Women with a diagnosis of DCIS or invasive breast cancer stages 0-III are allowed to participate and may be on endocrine therapy of any kind. If on endocrine therapy, women must be on endocrine therapy for 4 weeks and not expected to stop endocrine therapy during the study. If not on endocrine therapy, participants should not be planning to start during the course of the study. However, unexpected treatment with endocrine therapy will not require study withdrawal but will be recorded and considered in the analysis.

4.2 Exclusion Criteria

All candidates meeting any of the exclusion criteria at baseline will be excluded from study participation.

- Receiving other simultaneous treatment for hot flashes except antidepressants.
- Diagnosis of Major Depressive disorder or acute anxiety disorder in the past 5 years.
- Using any Complementary and Alternative Medicine (CAM) treatments for vasomotor symptoms. (This would include soy products and other phytoestrogens, black cohosh, and any mind-body techniques including meditation, yoga, etc.). If any therapies are used for reasons other than hot flashes and hot flash frequency if woman meets inclusion criteria, then woman is eligible)
- Stage IV breast cancer
- Serious psychological illness, specifically psychoses, schizophrenia or borderline personality disorder
- Currently using hypnosis for any reason
- PHQ-4 score ≥ 9
- Non-English speaking

4.3 Study Recruitment and Enrollment Procedures

Potential participants will be identified in three ways: (1) postcard mailings and invitation letters; (2) physician referrals and IRB approved chart screening; and (3) flyers, advertisements and press releases.

Physician referrals will come primarily from cancer centers at University of Michigan Cancer Center Breast Clinic in Ann Arbor, MI, and the Baylor Scott & White Health Center in Waco, TX.

At Michigan, key eligibility criteria will be used to identify potentially eligible women through the Data Office for Clinical and Translational Research (formerly the Honest Broker Office) and

Electronic Medical Record Search Engine (EMERSE). EMERSE (Electronic Medical Record Search Engine) provides a self-service web-based tool for authorized users to search clinical notes from our electronic medical record (CareWeb and Michart). Users can input their own terms or phrases and get results within seconds. The clinical notes include admission, discharge, and progress notes, as well as ambulatory care notes and notes from radiology, pathology, and other reports going back to 1998. With EMERSE, users can input a list of medical record numbers and search across that set, or can find a list of patients who have the term(s) of interest if it is not yet known who they are." Charts will be further screened, with IRB approval, for eligibility. If eligible, women will be sent an invitation letter where they can opt in or out of being contacted to learn more about the study. In addition, at Michigan, outreach will occur through obstetrics and gynecology, mammography, and internal medicine clinics to enable recruitment of women without a history of breast cancer. Mass mailings and local advertisement may also be used.

At Baylor University, a successful and proven method of accrual has been the use of mass mailings, local advertisement (e.g., newspaper, magazines, flyers). Additionally, Dr. Elkins is a medical associate with Baylor Scott & White Health Center, which provides a direct connection with physicians and medical staff who will provide potential referrals.

These research teams have a proven past in successful accrual for multiple research studies using similar recruitment techniques.

The targeted population for this study are women who wish to avoid hormonal therapy to treat hot flashes. Women who have gone through natural menopausal transition and those with a history of breast cancer will be included.

Those who are interested in participating in the study will be able to express their interest in doing so via phone, email, or text messaging. Research personnel will then correspond with these interested individuals via phone, email, or text messaging, to schedule a phone screening appointment.

During the phone screening appointment, more study details are provided. If the individual is still interested in participating after this information is provided, verbal consent will be obtained and potential eligibility will be determined using the Inclusion/Exclusion Criteria Screening Checklist which outlines the criteria listed in Sections 4.1 and 4.2. If a woman appears to be eligible, she will be scheduled for a baseline visit (conducted via telephone or video conference during COVID-19 restrictions) where the informed consent process for study procedures, the study purpose, procedures and requirements for baseline data collection will be explained. If she is interested in participating, she will sign the IRB-approved consent form to begin baseline data collection and be assigned a participant study ID number and will be considered enrolled in the study. If the baseline visit is conducted by telephone or video conference, the IRB-approved consent form will either be sent to the participant with a postage-paid envelope for return to the study team or sent through a secure, online platform. The online platform that will be utilized for this study will be DocuSign.

A Screening and Enrollment Log will be maintained at both sites to document eligibility/ineligibility, screen & consent dates, and referral source of each individual screened.

Once baseline data have been collected, a randomization/education visit (conducted by phone or video conference during COVID-19 restrictions) will be scheduled.

Eligible participants will be randomized to either self-administered hypnosis or self-administered sham white noise hypnosis. Randomization and mailing of appropriate intervention materials will take place several days before the visit if the visit is to take place through phone or video conference due to COVID-19 restrictions. Participants will also have an option of having the appropriate intervention materials shared with them electronically. Education about implementing their assigned arm will take place and for those assigned to hypnosis, the hypnotizability scale will be administered. However, the hypnotizability scale will not be administered if the randomization visit

takes place through phone or video conference due to COVID-19 restrictions. Randomization will be accomplished by the development of equally distributed tables of random numbers by the statistical team.

It is expected that each site (Baylor University and University of MI) will each recruit approximately half of the participants. Accrual will be closely monitored and efforts to randomize an equal number at each site will be made (see Section 9.4). Should accrual and randomization at either site significantly exceed 50% then the accrual percentage will be reduced at the other site accordingly. The randomization list will be kept by the study statistician.

5. STUDY INTERVENTIONS

5.1 Interventions, Administration, and Duration

5.1.1 Self-Administered Hypnosis Intervention

Participants in the self-administered hypnosis intervention group will be given booklets on the use of hypnosis for the treatment of hot flashes; and be provided with accompanying audio recordings containing hypnosis inductions as described above in Table 3. The booklets provide patient-education about hot flashes and treatment options, and include weekly instructions for a six-week long structured self-administered hypnosis intervention program for the treatment of hot flashes. A one-week baseline period will include the daily hot flash diary completion for the baseline week. During the baseline week, participants will also collect the physiologic measures, saliva three times per day over two consecutive days, and correspondingly, wearing the HRV monitor for 24 hours one day and for two 6 minute supine recordings on the other day. The physiologic measures will be optional for participants using phone or video conference for the baseline visit due to COVID-19 restrictions. Participants will then be asked to listen to audio-recordings of hypnosis inductions designed for the treatment of hot flashes, daily throughout 6 weeks. Each recording will be about 20 minutes long. Practices using the recordings will be documented on the Hypnosis Practice Log.

5.1.2 Self-Administered Sham White Noise Comparator

This group will serve as the control arm, but this hypothesis will be blinded to participants such that both arms will be introduced as self-administered hypnosis programs. This is a true statement that will avoid bias on the part of participants who may feel “cheated” if randomized to a control condition and could therefore bias the results. This arm has been developed to account for placebo and non-specific effects inherent in symptom management trials. Similar to the hypnosis group, this group will have a one-week baseline period that will include the daily hot flash diary completion for the baseline week. During the baseline week, participants will also collect the physiologic measures, saliva three times per day over two consecutive days, and correspondingly, wearing the HRV monitor for 24 hours one day and for two 6 minute supine recordings on the other day. The physiologic measures will be optional for participants using phone or video conference for the baseline visit due to COVID-19 restrictions. Participants will be given booklets with the same basic information on hot flashes, which includes education on the treatment options for hot flashes. This set of booklets will also be accompanied by an audio recording that contains information about hot flashes, and a 20-minute audio-recording of white noise. These audio-recordings provide no hypnotic induction, no guided relaxation, nor hypnotherapy. Participants will complete the baseline questionnaires at the baseline visit, with the exception of the hot flash daily diary which will be completed daily during the baseline week as in the hypnosis arm. Then participants will have a visit that will educate them about their randomized treatment and they will be asked to listen to their assigned

audio recording daily for 6 weeks.

This control condition is identical to that used in both Dr. Barton's and Dr. Elkins' previous research and matches the time, attention and modalities used in the hypnosis group.[39, 41] Participants were very satisfied with this arm in previous trials, had equal adherence as the hypnosis group and yet this arm yielded placebo effects equal to those obtained through a placebo pill in antidepressant trials. Participants assigned to this group will also be asked to listen to the audio recordings, focusing their attention in any way they feel would be helpful for reducing their hot flashes, and document their "practices" on the Hypnosis Practice Log.

This is an active control arm, accounting for all of the components in the intervention arm but lacking the mechanistic therapeutic approach and in previous research was shown to provide the same hot flash reduction as the placebo arm in other trials.[37, 74]

5.2 Handling of Study Interventions

Participants in both groups will be scheduled to receive phone calls from a research assistant approximately every 10 days, for a total of 5 phone calls during the six-week study period (see Table 2). The first phone call will be scheduled approximately 7 days after the randomization/education appointment. This call is intended to make sure the participant understands their assigned treatment. An additional phone call will be made during week 11 to remind the participant of the week 12 data collection.

All phone calls will be scripted and structured to allow research staff/participant-contact at scheduled intervals and may be as brief as a voice-mail left by the research staff providing call-back information to several minutes spent discussing general topics outlined on the Phone Call Checklist & Research Contact Staff Notes form. Research contact staff will document the calls using this form and participants will be encouraged to maintain and use the program materials; but there will be no therapeutic intervention during the call.

The delivery of the self-administered hypnosis intervention and the self-administered sham white noise hypnosis outlined in detail for both groups of the study in the Research Standardized Intervention Delivery Manual. Information regarding overall study procedures are detailed in the Manual of Procedures (MOP).

5.3 Concomitant Interventions

Concomitant interventions specifically for hot flashes are not allowed (see 4.1 and 4.2).

5.3.1 Allowed Interventions

Antidepressant use is allowed on study if the antidepressant has been for at least 2 months with no plans to change dose or antidepressant during this study and the participant meets the eligibility criteria of at least 4 hot flashes per day or 28 per week. Vitamin E is allowed. Any other treatments, if not used specifically for hot flashes, and participant meets hot flash frequency criteria, are allowed.

5.3.2 Required Interventions

None

5.3.3 Prohibited Interventions

See 5.3 and Inclusion/Exclusion Criteria (4.1 and 4.2)

5.4 Adherence Assessment

Adherence will be assessed through the frequency of at-home practice. Participants will complete the Self-Hypnosis Practice Log each day beginning at Week 1 and for the duration of their participation time in the study.

6. STUDY PROCEDURES

6.1 Schedule of Activities

**Table 4:** *Schedule of Activities*	**Enrollment**	**BL wk**	**Treatment and Follow-up in weeks**						
			**1**	**2**	**3**	**4**	**5**	**6**	**12**
Screening Checklist *including PHQ-4*	X								
Informed Consent	X								
Demographic Questionnaire & Health History Intake		X							
**<u>Aim 1 (primary outcome)</u>**									
Hot flash daily diary		D	D	D	D	D	D	D	D
**<u>Aim 2 (secondary outcomes)</u>**									
Hot Flash Related Daily Interference Scale		X						X	X
PROMIS Emotional Distress- Anxiety Scale		X						X	X
Pittsburgh Sleep Quality Index		X						X	X
Global Impression of change								X	
**<u>Aim 3 (mediators and moderators)</u>**									
Saliva collection for diurnal cortisol*		X						X	
HRV through Polar V800**		X						X	
Elkins Hypnotizability Scale (*only hypnosis group*)		X***							
Frequency of home practice			D	D	D	D	D	D	D
Check In Phone Calls			X	X	X	X	X		

Note: BL – Baseline data collection will occur; D – data collections occur daily completion over the course of the entire study through week 6, then again during week 12.

**samples will be collected over 2 days, upon awakening, 30 minutes later and a bedtime (around 10 pm); Note: this will be optional during COVID-19 restrictions*

***data will be collected during the same two days as cortisol sample collection; Note: this will be optional during COVID-19 restrictions*

****EHS will be done during randomization/education visit; Note: EHS-CF will be administered if visit is by telephone or video conference*

6.2 Description of Evaluations

The Schedule of Measures (**Table 4**) defines what is to be done at each week of the study period, which is approximately 13 weeks.

It is divided primarily by the type of evaluation (i.e.; Enrollment, Baseline, and Treatment & Follow-up). The Treatment & Follow-up period is further divided by study weeks.

6.2.1 Screening Evaluation

<u>Inclusion/Exclusion Criteria Screening Checklist</u>

This brief evaluation may be conducted over the phone and occurs to establish probable eligibility for the study. Questions are asked to confirm that the candidate meets the inclusion/exclusion criteria for the study, using a YES/NO format, and the 4-item PHQ-4 brief screening tool.

The PHQ-4 will be completed to assess exclusionary criteria of a PHQ-4 score ≥ 9. This brief screening measure was developed from the PHQ-9 and GAD-7 to be an ultra-brief screening tool for depression and anxiety disorders.[80] It has been validated in the general population and found to be comparable to the PHQ-8.[81] Scores on the PHQ-4 range from 0 to 12, with scores 0-2 being normal; 3-5 indicating mild symptoms; 6-8 moderate symptoms; and 9-12 severe. Cronbach's alpha for the 4 items was 0.85. The PHQ-4 was strongly correlated with the mental health subscale of the SF 20 (r=.80) and social functioning (r=.52). These correlation coefficients were nearly identical to those of the PHQ-8.[80]

Individuals scoring ≥9 on the PHQ-4 will be provided with additional referral information. At the University of Michigan, a referral will be made to psychology or psychiatry and the participant's oncologist will be notified, if applicable. There is also an on-call psychiatrist available Monday through Friday from 8 a.m. to 5 p.m. to assist with crisis situations. After hours, an on-call consultant can be reached by calling the clinic operator and arrangements can then be made to have the patient seen in the Emergency Room if necessary by the on-call psychiatrist. At Baylor University, a referral will be made to the Baylor Psychology Clinic (which is open Monday through Friday from 8 a.m. to 7:30 p.m.), Baylor Scott and White Health Center, or community medical health services. After hours, participants will be referred to the Emergency Room at Baylor Scott and White Health Center. (See Section 7.2).

Following an initial screen on the Inclusion/Exclusion Criteria Screening Checklist, participant candidates who meet the criteria from this checklist interview will be scheduled to attend the baseline appointment, where they will be asked to sign an informed consent form (ICF) prior to educational information about the study and/or baseline measures completion.

6.2.1.1 Consenting Procedure

There will be two consenting processes; one verbal consent process for the screening and a written informed consent process for study procedures.

Screening Verbal Consent

Research staff, not involved with study intervention will ask for verbal consent prior to completing the Inclusion/Exclusion Criteria Screening Checklist, using an IRB approved script either in person or over the phone with the candidate which states:

"Hello. My name is________and I am speaking with you in regard to a clinical trial involving the use of two different self-administered hypnosis programs for hot flashes that we are conducting at (Baylor University/University of Michigan). In order to complete the Inclusion/Exclusion Screening Checklist to assess your potential eligibility in this study, I will need to ask for your consent to participate to the screening process, which involves me asking and you answering questions that are all related to the criteria established to determine eligibility for participation in this study. The total time for the completion of this screening process is approximately 15 minutes…. Script is included in recruitment materials.

Individuals who respond with "NO" will be thanked for their time and the questions from the Inclusion/Exclusion Criteria Screening Checklist will not be asked.

The individual's response will be indicated by circling "YES" or "NO" on the script page and the screener will sign the form. These consent forms will be maintained with the Screening and Enrollment Log.

Written Informed Consent for Study Procedures

Research staff not involved with the study intervention will complete the informed consent process prior to beginning any study procedures.

Informed consent is a process that is initiated prior to the individual agreeing to participate in the study and continues throughout study participation. An IRB-approved, signed informed consent form (ICF) describing in detail the study procedures, risks/benefits, and participant rights will be given to the individual to follow during the consent process. The investigator or research staff will explain the research study to the participant using a standardized presentation format (e.g., PowerPoint presentation, written script), which will comprehensively cover all information in the ICF.

The individual will be encouraged to read through the document, or if requested, the ICF will be read aloud in its entirety. The individual may take the ICF to discuss with outside parties (e.g., spouse, friend, physician), or think about it prior to agreeing to participate, and have an opportunity to ask questions about the study. This informed consent form will include an addendum that addresses IRB-approved modes of communication throughout study participation (i.e., use of text messaging and email for scheduling purposes only). Once the ICF is signed by the participant and the person who explained the research study, the individual will be given a copy of the document, assigned a Study ID, and considered a participant in the study. Participants may withdraw consent at any time throughout the course of the study.

The consent process will be documented on the Data Collection Quality Control Form (DCQCF): Informed Consent and the signed original ICF will be filed separately from participant charts in a locked file drawer.

To complete the informed consent process at the end of study participation, study staff will inform the participant when his/her participation has come to an end and will document the discussion on the Data Collection Quality Control Form: Study Completion.

6.2.1.2 Screening

Screening is to identify eligible participants and will be completed during the completion

of the Inclusion/Exclusion Criteria Screening Checklist, after which individuals may be scheduled at any time to begin study participation, with the following exception:

- If at screening, the individual self-reports the use of other putative therapies for hot flashes for at least one month prior to enrollment, then a washout period of one month will be required prior to the scheduling of the baseline appointment.

6.2.2 Enrollment, Baseline, and/or Randomization

6.2.2.1 *Enrollment*

Once the candidate has met screening eligibility criteria, and signed the written ICF for study procedures, she will be considered enrolled in the study. Her enrollment date will be the date she signs the ICF. The participant's enrollment date will be recorded on the Enrollment Log. Participants will be enrolled during months 10 – 47 of the study. Not all participants who enroll will progress to randomization (due to screen failures and those who actively withdraw from the study), we anticipate enrolling individuals as needed at Baylor University to achieve our randomization goal of 131 participants randomized.

6.2.2.2 *Baseline Assessments*

Demographic Questionnaire & Health History Intake

The Demographic Questionnaire & Health History Intake is a brief intake questionnaire developed to capture participant characteristics, including age, race, ethnicity, education and similar demographics, time since first postmenopausal, history of breast cancer, and will be used to document no current use of alternative treatments for hot flashes.

Hot Flash Daily Diary

A Hot Flash Daily Diary will be used to measure the primary outcome of hot flash score. Participants will be asked to complete the Hot Flash Daily Diary every day for 6 weeks to measure the daily frequency and severity (mild, moderate, severe, and very severe) of hot flashes. Each level of severity is given a value; mild = 1; moderate = 2; severe =3; very severe = 4. Participates will rate the severity of each hot flash as they experience it and record it in their daily diary. A total hot flash score will be calculated for each participant (product of frequency x severity). For example, a woman with 3 mild and 2 severe hot flashes would have a hot flash score of 9 (3x1 = 3 + 2x3 =6). Participants are educated on how to collect hot flash information in real time throughout the course of each day. This process has been successfully used in numerous hot flash studies previously completed by Dr. Barton and colleagues[75] and other investigators including Dr. Elkins[38, 39] and has yielded replicable data in trials by different groups who evaluated the same treatments.[24] A hot flash diary will be provided to the participants at baseline and will be completed during the baseline week. A second diary will be provided for use during weeks 1 through 6 of the intervention period. A third hot flash diary will be used for week 12.

Hot Flash Related Daily Interference Scale (HFRDIS)

Participants will be asked to complete the Hot Flash Related Daily Interference Scale (HFRDIS) during baseline and weeks 6 and 12. This is a 10 item scale that measures hot flash interference.[82] The HFRDIS asks respondents to rate the degree on a 0-10 scale that the hot flashes interfere with various daily activities as well as overall enjoyment or quality of life. This measure has been shown to be valid and internally consistent, with an alpha of .96.[82] This will be completed at baseline, 6 and 12 weeks.

PROMIS™ Emotional Distress – Anxiety Scale

The PROMIS™ Emotional Distress – Anxiety Scale contains 29 items that measure 5 domains of anxiety: 1) affective, 2) cognitive, 3) somatic, 4) behavioral, and 5) need for treatment. Items are scored on a scale from 1-5 with higher scores reflecting more severe anxiety. Like all PROMIS measures, the anxiety scale was developed through rigorous qualitative and quantitative assessment involving expert review, focus groups, and item response theory analysis, and it has shown excellent convergent and discriminate validity with other measures, with a Cronbach's alpha of .95.[83, 84] This will be completed at baseline, 6 and 12 weeks.

Pittsburg Sleep Quality Index (PSQI)

The PSQI is a 19-item self-report inventory designed to measure sleep disturbance. The 19 items are grouped into 7 subscales: 1) sleep quality; 2) sleep efficiency; 3) daytime dysfunction; 4) sleep latency; 5) sleep disturbances; 6) sleep duration; and 7) use of sleep medication. These seven subscales are scored on a scale of 0-3 with higher scores indicating greater sleep pathology. Previous studies indicate that PSQI total scores are significantly correlated with measures of sleep onset latency, amount of time spent awake after initial sleep onset, and total sleep time as assessed by sleep diary and wrist actigraphy,[85] and alphas for the PSQI range from .70 to .80.[86] A cut point of 5 has been determined through evidence to differentiate sleep disturbance (≤5) from normal sleep.[87] This will be completed at baseline, 6 and 12 weeks.

Subject Global Impression of Change The Subject Global Impression of Change is a 7-point item in which the participant rates the change in hot flashes since beginning the study (ranging from "very much better," "moderately better," "a little better," "about the same," "a little worse," "moderately worse," to "very much worse"). It has been used extensively for determination of minimally clinically significant differences in numerous oncology clinical trials.[88, 89] Four additional investigator-developed questions will be included, of those four, two will ask about satisfaction . This will be completed only at 6 weeks. In addition, two questions about the use of audio recordings are included.

Diurnal Cortisol Rhythm

Each participant will be provided a kit with salivettes for the collection of saliva. Saliva will be collected three times a day over a two consecutive day period at baseline and 6 weeks. Participants will be provided specific directions for the collection of saliva upon awakening, (before getting out of bed), 30 minutes later (before eating, drinking or tooth brushing), and finally at bedtime (around 10 pm, also before tooth brushing) and will be given a Saliva Collection Diary with questions to answer surrounding their saliva collection. Participants will also be instructed to complete Daily Health Behaviors questionnaires during the same two-day period. (This procedure is well established and is considered valid for the measurement of diurnal cortisol rhythm.)[67, 68]

Cortisol collection is explained in the baseline visit. During the COVID-19 restrictions, this interaction will occur by telephone or video conference. If the participant is unable to demonstrate understanding of the collection process, we will not collect cortisol on that participant in the study. Additionally, if there is any interruption to mail service or to the lab where specimens are banked, this portion of the study will be paused during COVID-19 restrictions.

Measures of Heart Rate Variability

The shift in autonomic balance from sympathetic dominance (stress response) to parasympathetic dominance (relaxation response) is a very likely candidate for the

underlying mechanism of the effects of mind-body interventions on vasomotor symptoms. Relatedly, an increasing body of evidence indicates that hot flash events are associated with changes in heart rate variability.[90-92] The Polar V800, a mobile HRV recording device will be worn to collect data over a 24-hour natural ambulatory period as well as two 6 minute supine recordings during the two days of cortisol collection at baseline and 6 weeks. The 24 hr and two 6 minute measurements will be completed (each on separate days) at pre- and post-trial. The Polar V800 has been shown to have equivalency for the time domain of HRV.[93]

The ambulatory device will be returned to the investigators in a pre-posted mailer immediately following each HRV data collection (i.e., baseline and week 6 endpoint). Data will be downloaded as soon as it is received in the lab, for optimal resolution and to protect privacy. The device will then be cleared of data and available for the next use, which should ensure that study recruitment is not delayed due to a shortage of Polar V800 devices.

Heart rate variability monitoring is explained in the baseline visit. During the COVID-19 restrictions, this interaction will occur by telephone or video conference. If the participant is unable to demonstrate understanding of the collection process, we will not collect heart rate variability on that participant in the study. Additionally, if there is any interruption to mail service, this portion of the study will be paused during COVID-19 restrictions.

Perceived Stress Scale (PSS)

The PSS contains 10 items that measure the degree to which situations in one's life are appraised as stressful.[94] The scale provides the degree to which individuals find their lives to be unpredictable, uncontrollable, and overloaded, as well as measures the current levels of experienced stress.[95] Items are scored on a scale from 0-4 with higher scores reflecting more perceived stress. This measure has been shown to be valid and internally consistent, with an alpha of .83.[94] This will be completed at baseline, 6, and 12 weeks.

Elkins Hypnotizability Scale (EHS)

The EHS is a 12-item, research assistant-administered, scale for rating hypnotizability in the general population. Each item is rated pass\fail, with higher scores indicative of greater hypnotizability.

Previous research indicates that the EHS is highly correlated with longer measures of hypnotizability and has excellent reliability with a Cronbach's alpha of .94.[96] This will be administered to participants randomized to the hypnotic intervention group at the randomization visit. The Elkins Hypnotizability Scale-Clinical Form (EHS-CF) will be administered during the randomization visit when the randomization visit takes place through telephone or video conference because of COVID-19 restrictions. The EHS-CF a less intensive and shorter version with 9 items compared to the full 12. However, this is not a different scale and provides the same measures we are seeking with a high correlation of .962 between the EHS and EHS-CF. The EHS-CF will allow for an easier implementation of the scale through remote procedures and be more user-friendly for the participants.

Self-Hypnosis Practice Log

Frequency of home practice is thought to be one of the moderators of the *efficacy* of hypnosis interventions[70] and will be particularly important to collect for this self-administered protocol.[70] Participants in both groups will be given a log that they will complete daily. The log will provide space to put the date, whether, or not the participant listened to their audio recording, the number of times they practiced (with and without the audio recordings), and

provide a space for comments about any interruptions or barriers to listening to their assigned recording. Between the log and the phone calls, the research assistant will be able to ascertain the degree to which women adhered to the protocol in terms of recording hot flashes in real time and using the audio-recordings as prescribed in the protocol. This will be completed daily during weeks 1 to 6 of the intervention and during week 12.

6.2.2.3 Randomization

Randomization will be assigned after baseline measures have been completed and prior to intervention instruction and administration.

If a period of more than 30 days passes after the screen is complete without the individual coming to the lab for the baseline appointment (except in the case of washout of putative therapies for hot flashes for one month prior to enrollment on study), the initial verbal consent will be considered void, and will need to be repeated and another Inclusion/Exclusion Criteria Screen Checklist will be completed prior to rescheduling the baseline visit. Since randomization will not have taken place, this will not impact accrual numbers but will be counted as a screen failure. Participants who attend the baseline visit and sign the consent form but then never return for their randomization visit will also be considered screen failures, but these women will be replaced to maintain statistical power.

Once randomization has been completed, efforts will be made to ensure that participants enrolled are assisted and encouraged to initiate and complete participation in the materials associated with the condition to which they are assigned.

6.2.3 Blinding

In order to reduce the chance for bias, research staff implementing this study will either be blinded to treatment assignment or not. Blinded staff will be involved in activities that will ensure those individuals remain blinded throughout the study. Unblinded staff will perform activities that will require knowledge of the participants study arm. The specific roles and personnel are listed in the Delegation of Authority Log.

PIs, data collectors and the study statistician will remain blind to group allocation until the database is locked. PIs and research staff will not have access to the randomization list. Blinding of all study research assistants is not possible with this study design, as they will be interacting with participants via phone calls and the nature of the discussion may disclose the type of recordings the participant is using (i.e., white noise or hypnosis intervention with induction). Approximately one to three staff members will be unblinded and perform the randomization, education and follow up phone calls. The data is self-reported by the participants and will be mailed back to the research site, where an unblinded research staff member will receive it upon completion.

A limited number of research staff will be identified on the Delegation of Authority Log as **unblinded research staff**, and will be authorized to:

- Inform of randomization assignment
- Administer the EHS
- Provide initial education regarding implementing assigned arm
- Provide telephone follow up calls
- Prepare required quarterly/annual reports to NCCIH, DSMB, and IRB
- Other tasks as delegated in the Delegation of Authority log

Participants will also be blinded to the study hypotheses to avoid bias regarding what is the experimental treatment versus control arm. After the 12-week data point, participants in the self-administered sham white noise hypnosis arm who have completed the study and returned all data will be offered the set of hypnosis audiotapes.

6.2.3 Follow-up Visits

Week 1-6: Eligible participants will be randomly allocated to either a self-administered hypnosis group or a self-administered sham white noise hypnosis control group. Both groups will meet with a study research assistant two times (at baseline and at randomization/education visit) in person (or by telephone or video conference during COVID-19 restrictions) and further contact will be by phone through week 6 of the intervention period.

The interventions will be delivered through pre-recorded digital audio files, flash drives or CD's. For participants who prefer a flash drive or CDs, those will be provided. Additionally, if a participant prefers to use CDs, but does not have a CD player, one will be provided. Prepared self-management booklets will also be provided to each participant.

Participants in the self-administered hypnosis group and the self-administered sham white noise hypnosis group will use the self-management materials (booklets and audio recordings) for six weeks. A second kit to collect saliva and a Polar V800 HRV monitor will be mailed to participants during week five to be used for Week 6 data collection. At the end of Week 6, they will collect two days of saliva samples. HRV data will also be collected during the two days of saliva collection (as done during the baseline week) and both will be returned immediately following collection via a pre-posted mailer. However, if this collection was optional or unable to be completed due to COVID-19 restrictions, saliva samples and HRV data will not be collected at this time. At the end of Week 6, participants will return the completed hot-flash daily diaries, the self-hypnosis practice log, the questionnaire booklet with outcome measures of daily activity interference, quality of life, sleep disturbance, anxiety and stress in the provided pre-posted mailer. Due to COVID-19 restrictions, participants will also have the option of returning materials electronically (e.g., through DocuSign).

6.2.4 Completion/Final Evaluation

Week 12: Before the start of week 12, participants will be telephoned to remind them of the 12 week data collection. The diary, log and questionnaires to be completed that week are included in the toolkit. At the end of week 12, participants will return the completed hot-flash diaries, self-hypnosis practice log, and complete questionnaire booklet that includes the measures of daily activity interference, quality of life, sleep disturbance, anxiety, stress and impression of change in the provided pre-posted mailer. Due to COVID-19 restrictions, participants will also have the option of returning materials electronically (e.g., through DocuSign).

Upon receipt of these measures, the Data Collection Quality Control Form: Study Completion will be completed, signed by the CRC, and countersigned by the PI.

Participants who complete the baseline and 6 week data collection will receive payment (gift card or check) in the amount of $75 and if they complete the 12 week data collection, they will receive another payment (gift card or check) in the amount of $50 to maximize retention for the longer data collection efforts.

7. SAFETY ASSESSMENTS

Study progress and safety will be reviewed monthly (and more frequently if needed). Progress reports, including patient recruitment, retention/attrition, and AEs will be provided to the DSMB every six months. A report will be compiled and will include a list and summary of AEs. In addition, this report will address (1) whether AE rates are consistent with pre-study assumptions; (2) reason for dropouts from the study; (3) whether all participants met entry criteria; (4) whether continuation of the study is justified on the basis that additional data are needed to accomplish the stated aims of the study; and (5) conditions whereby the study might be terminated prematurely. The report will be provided to the Board members and will be forwarded to the IRB; institution-based DSMBs as required by the institution; and NCCIH along with a letter of recommendation from the Board Chair. The IRB, NCCIH, and other applicable recipients will review progress of this study on an annual basis

7.1 Specification of Safety Parameters

We are excluding women around mental health diagnoses and screening with the PHQ-4 that would make hypnosis a potentially unsafe intervention.

7.2 Methods and Timing for Assessing, Recording, and Analyzing Safety Parameters

Expected risks are minimal. However, plans to address potential risks to the participant are as follows:

- Anxiety: Severe or acute anxiety or agitation will be evaluated pre-study during the history intake, as well as assessed through phone follow ups. The main risk related to this study is increased anxiety from a sense of worry about whether one is performing the assigned treatment arm correctly. Negative side effects have not been realized in any of the previous studies Drs. Elkins and Barton have completed with hypnosis, including the structured control and white noise arms.[38, 41] In fact, in all of those studies, participants in both arms generally improved in most aspects of health-related quality of life such as anxiety, agitation and feelings of well-being. Any evaluation of developing issues with acute anxiety or distress, as evaluated by research assistants who will be contacting participants throughout the study, will be immediately brought to the attention of the PI's at Baylor and the University of Michigan. At the University of Michigan, a referral will be made to psychology or psychiatry. There is also an on-call psychiatrist available Monday through Friday from 8 a.m. to 5 p.m. to assist with crisis situations. After hours, an on-call consultant can be reached by calling the clinic operator and arrangements can then be made to have the patient seen in the Emergency Room if necessary by the on-call psychiatrist. At Baylor University, a referral will be made to the Baylor Psychology Clinic which is open Monday through Friday from 8 a.m. to 7:30 p.m. After hours, participants will be referred to the Emergency Room at Baylor Scott and White Health Center. Research assistant staff are trained in all study procedures, and work directly under the supervision of Dr. Elkins, PhD, and Dr. Barton, RN, PhD, FAAN. Measures will be taken to ensure each woman understands her right to withdraw from the study at any time for any reason. If the research assistant assesses, at any time, that study participation is causing increased distress or an undue burden on a participant, they will address this issue and again assure the woman of her right to withdraw.
- Emotional distress: While there does exist the risk of emotional distress, the risk is rare with the type of hypnotic induction used in this study. If participants score 9 or over on the PHQ-4, they will be referred to their provider immediately for care. Participants will be advised during the consent process that this referral may be required. Participants will be advised of this as a risk, and any participant who experiences emotional distress will be given the opportunity to discuss

her feelings with the PI, and if needed, further referrals will be made.

- HRV Recording device: The Polar V800, a mobile HRV recording device is a non-significant risk device if the protocol’s exclusionary criteria are adhered to. Wearing the device can be a minor inconvenience, comparable to that of a standard wrist watch. The device poses no risk of electric shock. There are no known potential toxicities, however there could be skin irritation caused by the band that goes around the wrist and chest.
- Loss of confidentiality: there is a small risk that confidential information could be seen by someone not directly involved in the study activities. The investigators have developed critical processes to minimize this risk including research coding that does not include PHI, locked files, and encrypted and firewall protected databases.

These risks are considered to be minimal and are described in the protocol and consent form.

Steps will be taken to minimize any potential risks associated with study participation. Participation in this research study is strictly voluntary. The participant may opt to withdraw consent at any time for any reason. The investigator will notify the participant of any new information related to risk, toxicity, or efficacy that could influence the decision by the participant or investigator to continue study therapy.

7.3 Adverse Events and Serious Adverse Events

An **adverse event (AE)** is generally defined as any unfavorable and unintended diagnosis, symptom, sign (including an abnormal laboratory finding), syndrome or disease which either occurs during the study, having been absent at baseline, or if present at baseline, appears to worsen. Adverse events are to be recorded regardless of their relationship to the study intervention.

A **serious adverse event (SAE)** is generally defined as any untoward medical occurrence that results in death, is life threatening, requires inpatient hospitalization or prolongation of existing hospitalization, results in persistent or significant disability/incapacity, or is a congenital anomaly.

Adverse events (AEs) and serious adverse events (SAEs) that are unanticipated, serious, and possibly related to the study intervention will be reported to the DSMB, IRB, and NCCIH in accordance with requirements.

Unexpected fatal or life-threatening AEs related to the intervention will be reported to the NCCIH Program Officer within 7 days. Other serious and unexpected AEs related to the intervention will be reported to the NCCIH Program Official within 15 days.

Anticipated or unrelated SAEs will be handled in a less urgent manner but will be reported to the DSMB, IRB, and NCCIH in accordance with the respective requirements.

Information to be reviewed at the DSMB meetings includes recruitment, accrual and dropout statistics, number and description of any AE or SAE, comparison of AE and SAE to stop rule for determination of need for study closure, and any other additional concerns. In the annual summary, the DSMB Report will state that they have reviewed all AE and SAE reports.

During the intervention period, at each phone contact by the research staff, participants will be assessed using the CTCAE grading scale for agitation, anxiety, and skin rash.

7.4 Reporting Procedures

AEs will be collected and recorded as illustrated on **Table 5**, which includes the Participant ID, a brief description of the AE, AE start date, AE type, AE intensity, study attribution of the AE, PI initials, and date initialed.

Table 5: *Adverse Event Reporting Log*

**STUDY NAME:**							
**Principal Investigator:**<br>**Study Coordinator:**				**Site:**			
Adverse Events: ☐ None							
Participant ID	Brief Description of AE	Start Date (mm/dd/yy)	Type of AE	AE Intensity	Study Attribution	PI Initials	Dated (mm/dd/yy)
			☐ Expected<br>☐ Unexpected	☐ 1: Mild<br>☐ 2: Moderate<br>☐ 3: Severe<br>☐ 4: Life Threatening/Disabling<br>☐ 5: Death	☐ Unrelated<br>☐ Unlikely<br>☐ Possible<br>☐ Probable<br>☐ Definite		
			☐ Expected<br>☐ Unexpected	☐ 1: Mild<br>☐ 2: Moderate<br>☐ 3: Severe<br>☐ 4: Life Threatening/Disabling<br>☐ 5: Death	☐ Unrelated<br>☐ Unlikely<br>☐ Possible<br>☐ Probable<br>☐ Definite		

Any expected or unexpected grade 3 or 4 adverse events with at least an attribution of possibly will be reported, with serious AE's reported in 24 hours to IRB and NCCIH.

7.5 Follow-up for Adverse Events

Participants experiencing a grade 3 or 4 AE that is at least possibly related will be withdrawn from the study.

7.6 Safety Monitoring

The Data Safety Monitoring Board for this study is comprised of individuals with expertise in biostatistics, clinical research, and clinical aspects of the patient population not associated directly with recruitment, consent, intervention delivery or data management, and who work independently of the Co-PIs, Dr. Gary Elkins and Dr. Debra Barton and Co-Investigators, Dr. Grant Morgan, Dr. Tierney Lorenz, and Dr. Catherine Van Poznak. They are not part of the key personnel involved in this grant. No member of the Board will have co-published with the either of the Co-PIs within the past three years. Board members are qualified to review the patient safety data generated by this study because of their unique expertise relative to this study. The CVs of all members of the DSMB are included in the Data Safety and Monitoring Plan (DSMP) and will be updated as needed.

8. INTERVENTION DISCONTINUATION

During intervention delivery, participants will be closely monitored, and the following "stopping rule" will apply.

For an individual participant, all study procedures will be stopped if:

- the participant wishes to stop participation and/or,
- a serious and/or unexpected grade 3 (serious) or 4 (life-threatening) AE occurs with an attribution of at least possibly related.

With their permission, randomized participants will continue to be followed if the study intervention is discontinued until the end of what would have been their participation period (i.e., to the 12-week assessment). These procedures will ensure the safety of the participants.

Additionally, the study may be closed to participant enrollment based on careful consideration of

adverse events or recruitment issues as determined by the DSMB. (See Section 9.4)

9. STATISTICAL CONSIDERATIONS

General Design Issues

9.1 Primary Objective

The primary objective is to fully evaluate the efficacy of the self-administered hypnosis intervention for hot flashes, with the indication that self-administered hypnosis will significantly decrease vasomotor symptoms compared to the self-administered sham white noise hypnosis group. In order to obtain an estimate of the efficacy of the self-administered hypnosis that is not positively biased, the reported hot flashes will be analyzed using intent-to-treatment analysis.

Hypothesis 1.1

Self-administered hypnosis will significantly decrease vasomotor symptoms (hot flash score of frequency and severity) compared to the self-administered sham white noise hypnosis at 6 weeks.

Outcome Measure:

- Hot Flash Daily Diary

9.2 Secondary Objectives

Secondary objectives are to evaluate the efficacy of the self-administered hypnosis compared to self-administered sham white noise hypnosis for hot flash activity interference, sleep disturbance, anxiety and perception of benefit; with the expectation that there will be significant improvements among participants in the hypnosis group when compared with the self-administered sham white noise hypnosis group. Further, we will evaluate the effects of self-administered hypnosis compared to the self-administered sham white noise hypnosis control at 12 weeks. In order to obtain estimates of the efficacy of the self-administered hypnosis that is not positively biased on the secondary outcome measures, each secondary outcome measure will be analyzed using intent-to-treatment analysis

Hypothesis 2.1

Compared to self-administered sham white noise hypnosis, self-administered hypnosis will result in significant improvements in hot flash activity interference, sleep disturbance, and anxiety at 6 and 12 weeks and perception of benefit at 6 weeks.

Outcome Measures:

- Hot Flash Related Daily Interference Scale
- PROMIS Emotional Distress – Anxiety Scale
- Pittsburg Sleep Quality Index
- Subject Global Impression of Change

Additionally, mediators (perceived stress, cortisol, and heart rate variability (HRV) and moderators (hypnotizability and practice adherence) of hot flash reduction will be explored to address the following hypotheses:

Hypothesis 2.2

A decrease in perceived stress will be indicated in the self-administered hypnosis group due to the intervention, which will correspond and contribute to further decrease of the hot flash score at 6 and 12 weeks.

Outcome Measure:

- Perceived Stress Scale

Hypothesis 2.3

Diurnal salivary cortisol will demonstrate better circadian rhythms (steeper slopes) in those with larger hot flash reductions and in the self-administered hypnosis group compared to self-administered sham white noise hypnosis at 6 weeks.
Outcome Measure:

- Diurnal Cortisol Rhythm

Hypothesis 2.4

Self-administration of hypnosis and greater hot flash reductions will result in higher resting and 24-hour parasympathetic activity, reflected as higher HRV, compared to self-administered sham white noise hypnosis at 6 weeks.
Outcome Measure:

- Heart rate variability monitor

Hypothesis 2.5

Higher hypnotizability scores and better adherence rates will be associated with greater reductions in hot flash scores in the self-administered hypnosis group.
Outcome Measures:

- Self-Hypnosis Practice Log
- Elkins Hypnotizability Scale

Hypothesis 2.6

Self-administered hypnosis will significantly decrease vasomotor symptoms (hot flash score of frequency and severity) compared to the self-administered sham white noise hypnosis at 12 weeks.
Outcome Measure

- Hot Flash Daily Diary

9.3 Sample size and randomization

We estimated the required sample size for this study with an a priori power calculation. Data from our previous studies were used to inform the power calculations.[38, 39] The daily hot flash entries are aggregated every seven days to create a weekly hot flash score. As a result, there will be one hot flash score at baseline and one for each week of data collection. For Aim #1, seven hot flash scores will be used (i.e., baseline score + weekly score for weeks 1 through 6) for each group. For this aim, we will evaluate whether or not average weekly hot flash scores between groups follow the same change pattern across time. This effect is the group-by-time interaction effect. We initially considered the G*Power software,[97] but opted for the Optimal Design software[98] because it is more readily capable of providing power estimates for designs that involve multilevel data; that is, longitudinal data (e.g., repeated measures) and/or hierarchically structured (i.e., nested) data. Data from our previous studies were used to specify the relative expected amount of variability attributable to the participants, including group differences, and the relative expected amount of variability due to natural fluctuation of each person's scores over time. A secondary benefit of using a multilevel framework for estimating power in this study is that the sample size necessary for addressing the secondary objectives are built into the sample size calculation for objective 1.

Our previous studies reveal that the vast majority of the variability in hot flash scores, including changes over time, is explained by differences between people (e.g., group assignment) rather than hot flash score simply varying to any meaningful extent for any given person across weeks. Therefore, we specified, realistically, that nearly 80% of the variability in hot flash scores across time will be attributable to differences between people. In our preliminary data mean and standard deviations for hot flash score in the intervention group at baseline was 23.43 (1.02) and follow-up at week 12; 4.61 (0.56); means and standard deviations for the control arm at baseline were 22.94 (1.23) and follow-up at week 12, 19.41 (1.36). The mean reduction for hot flash score was 18.83 for the hypnosis group from baseline as compared with 3.53 in the controls ($p < .0.001$; 95% CI, 12.60-17.54). Clinically significant improvement is considered to be 50% reduction over baseline.[37] We expect, at minimum, a 50% reduction in hot flash scores in the self-administered hypnosis group and 25% reduction in the self-administered sham white noise hypnosis group.

Based on previous studies, the mean hot flash score for post-menopausal women self-administering hypnosis is about 23 with standard deviation of 10.9. We simulated 10,000 data points from a normal distribution with mean of 23 and standard deviation of 7; we used a smaller standard deviation because the standard deviation from the previous pilot was based on a small sample. Next, we simulated a 50% reduction in hot flash scores for the 5,000 simulated values to mimic the expected effect self-administered hypnosis post treatment. We also simulated a 25% reduction in hot flash scores for the other 5,000 simulated values to mimic the expected effect of the self-administered sham white noise hypnosis at the post treatment data point. The hot flash distribution for the simulated self-administered hypnosis condition at the post treatment data point had mean of 11.4 (SD=9.8) whereas the distribution for the simulated structured attention condition at post treatment had a mean of 17.2 (SD=9.9). These effects represent a strictly standardized mean difference of .41 between the self-administered hypnosis and structured attention conditions. We used the strictly standardized mean difference in lieu of the traditional standardized mean difference because it is slightly more conservative to ensure sufficient power. The strictly standardized mean difference in the reduction rates, when taking group variability into account, of 0.41 was entered into G*Power and Optimal Design software. Using a Type I error rate (α) of 5% and power of 80%, the required sample size is 190 without attrition in both power analysis programs. Because the within-between interaction for two time points (i.e., baseline and follow-up) is equivalent to an independent samples t test on the difference scores, Power was computed in the G*Power using the t distribution for independent samples on the difference scores. We entered the effect size of .41, Type I error rate of 5%, Power of 80%, and N2/N1 ratio of 1 (i.e., balanced design). The required sample size was 190. Power was cross-validated in the Optimal Design software using the Person Randomized Trial Design with Single Level Trials (i.e., participants are the experimental units). Using Type I error rate of 5% and effect size of .41, the required sample size was 190. Given the expected attrition rate of 18% attrition or less, the sample size required to accomplish all aims of this study is 232 participants. We should note that the framework described above for determining power generalizes to objective #2 and the moderator analysis described in objective #3. The power analysis should also be sufficient for the mediation analysis.

Treatment Assignment Procedures

Enrolled participants will be randomized to either self-administered hypnosis or self-administered sham white noise hypnosis. Randomization will be accomplished by the statistical team with the development of two case assignment spreadsheets; one for each site, thus assuring equal treatment arms by site. Randomization will be accomplished by the statistical team using stratified permuted block randomization with fixed blocks of size four. Two strata will be used: site (University of Michigan or Baylor University) and participant subgroup (post-menopausal or breast

cancer). All randomization block permutations will be generated, and one permutation will be randomly assigned to each block. Given that block size will be fixed at four, there are six permutations of randomization. One of the six permutations will be randomly to each block of participants. The randomization information will be printed on individual cards (e.g., “46BRG32: Self-Administered Hypnosis Group” or “46BRG32: Self-Administered Sham White Noise Hypnosis Group”), each of which will be placed in a brown security envelope and sealed (one card per envelope). It is expected that each site (Baylor University and University of MI) will each randomize approximately half of the participants, but each site will receive two sets of 116 envelopes – one set of 116 for each participant subgroup. It is not expected that there be an equal distribution of participants who are post- menopausal or breast cancer survivors; rather, the randomization strategy ensures an equal distribution of each subgroup into the self-administered hypnosis intervention or self-administered sham white noise hypnosis condition regardless of the proportion of each participant subgroup within site. For example, if Baylor University randomizes 100 participants who are post-menopausal and 16 who are breast cancer survivors, having two sets of 116 randomization envelopes ensures that (1) there are enough envelopes and (2) there are 50 post-menopausal and 8 breast cancer survivors in each condition. Therefore, one set of sealed envelopes for post-menopausal participants with group assignment and one set of sealed envelopes for breast cancer survivors with group assignment will be provided to both Dr. Elkins’ team and Dr. Barton’s team. The case assignment spreadsheets will be kept by the statistician, and not accessible to persons who will be educating, obtaining consent from the participants, collecting data, or interacting in any other capacity which involves contact with participants, as this may possibly cause bias in the recruitment process. All participants randomized will be included in the analyses (i.e., intent-to-treat analysis).

9.4 Definition of Populations

The population will be 232 postmenopausal women, age 18 or older. For more detailed description of the study population see *Section 4. SELECTION AND ENROLLMENT OF PARTICIPANTS.*

9.5 Interim Analyses and Stopping Rules

No interim analyses of primary or secondary data are anticipated.

This study will be stopped prior to its completion if:

- In the opinion of the DSMB and IRB, the intervention is associated with adverse effects that call into question the safety of the intervention.
- If there are life-threatening SAEs that are related or potentially related to the study intervention the DSMB would discuss the risk of the intervention to the subjects and make a recommendation on continuation of the study.
- Recruitment or retention becomes untenable to the extent that study end point data collection is not possible.
    - The two PIs will meet at least monthly (and more frequently if needed) to review accrual goals. Should the situation arise that one site recruits more quickly than the second site (to the extent that the ability to meet accrual goals appear endangered), the Co-PIs will confer to increase accrual efforts at that site. Should these actions fail to produce the anticipated and necessary results for the site, the Co-PIs will confer with the NCCIH Program Officer (PO) and DSMB to request approval for increased accrual at the other site.

- New information becomes available during the trial that necessitates stopping the trial;
- Other unanticipated problems or situations occur that might warrant the IRB, DSMB, or NCCIH stopping the trial.

9.6 Outcomes

9.6.1 Primary Outcome

Hot flash score: Hot Flash Daily Diary

A Hot Flash Daily Diary will be used to measure the primary outcome of hot flash score. Participants will be asked to complete the Hot Flash Daily Diary every day for 6 weeks to measure the daily frequency and severity (mild, moderate, severe, and very severe) of hot flashes. Each level of severity is given a value; mild = 1; moderate = 2; severe =3; very severe = 4. Participants will rate the severity of each hot flash as they experience it and record it in their daily diary. A total hot flash score will be calculated for each participant (product of frequency x severity). For example, a woman with 3 mild and 2 severe hot flashes would have a hot flash score of 9 (3x1 = 3 + 2x3 =6). Participants are educated on how to collect hot flash information in real time throughout the course of each day.

9.6.2 Secondary Outcomes

Hot flash activity interference: Hot Flash Related Daily Interference Scale (HFRDIS)

Participants will be asked to complete the Hot Flash Related Daily Interference Scale (HFRDIS) during baseline and weeks 6 and 12. This is a 10 item scale that measures hot flash interference.[82] The HFRDIS asks respondents to rate the degree on a 0-10 scale that the hot flashes interfere with various daily activities as well as overall enjoyment or quality of life. This measure has been shown to be valid and internally consistent, with an alpha of .96.[82] Individual items and a total summary score will be used.

Anxiety: PROMIS™ Emotional Distress – Anxiety Scale

The PROMIS™ Emotional Distress – Anxiety Scale contains 29 items that measure 5 domains of anxiety: 1) affective, 2) cognitive, 3) somatic, 4) behavioral, and 5) need for treatment. Items are scored on a scale from 1-5 with higher scores reflecting more severe anxiety. Like all PROMIS measures, the anxiety scale was developed through rigorous qualitative and quantitative assessment involving expert review, focus groups, and item response theory analysis, and it has shown excellent convergent and discriminate validity with other measures, with a Cronbach's alpha of .95.[83, 84]

Sleep disturbance: Pittsburg Sleep Quality Index (PSQI)

The PSQI is a 19-item self-report inventory designed to measure sleep quality. The 19 items are grouped into 7 subscales: 1) sleep quality; 2) sleep efficiency; 3) daytime dysfunction; 4) sleep latency; 5) sleep disturbances; 6) sleep duration; and 7) use of sleep medication. These seven subscales are scored on a scale of 0-3 with higher scores indicating greater sleep pathology. Previous studies indicate that PSQI total scores are significantly correlated with measures of sleep onset latency, amount of time spent awake after initial sleep onset, and total sleep time as assessed by sleep diary and wrist actigraphy,[85] and alphas for the PSQI range from .70 to .80.[86] A cut point of 5 has been determined through evidence to differentiate sleep disturbance (≤5) from normal sleep.[87]

Perception of benefit: Subject Global Impression of Change

The Subject Global Impression of Change is a 7-point item in which the participant rates the change in hot flashes since beginning the study (ranging from "very much better," "moderately better," "a little better," "about the same," "a little worse," "moderately worse," to "very much

worse"). It has been used extensively for determination of minimally clinically significant differences in numerous oncology clinical trials.[88, 89] Four additional investigator-developed questions will be included, of those four, two will be about satisfaction..

Diurnal Cortisol Rhythm

Each participant will be provided a kit with salivettes for the collection of saliva. Saliva will be collected three times a day over a two-day period. Participants will be provided specific directions for the collection of saliva upon awakening, (before getting out of bed), 30 minutes later (before eating, drinking or tooth brushing), and finally at bedtime (around 10 pm, also before tooth brushing) and will be given a Saliva Collection Diary with questions to answer surrounding their saliva collection. (This procedure is well established and is considered valid for the measurement of diurnal cortisol rhythm.[67, 68]

Measures of Heart Rate Variability

The shift in autonomic balance from sympathetic dominance (stress response) to parasympathetic dominance (relaxation response) is a very likely candidate for the underlying mechanism of the effects of mind-body interventions on vasomotor symptoms. Relatedly, an increasing body of evidence indicates that hot flash events are associated with changes in heart rate variability**.**[90-92] The Polar V800, a mobile HRV recording device will be worn to collect data over a 24-hour natural ambulatory period as well as two supine standardized 6-minute recordings. The 24-hr and 6 min measurements will be completed (each on separate days) at pre- and post-trial. The ambulatory device will be returned to the investigators and downloaded for optimal resolution and to protect privacy. The Polar V800 has been shown to have equivalency for the time domain of HRV.[93]

Perceived Stress Scale (PSS)

The PSS contains 10 items that measure the degree to which situations in one's life are appraised as stressful.[94] The scale provides the degree to which individuals find their lives to be

unpredictable, uncontrollable, and overloaded, as well as measures the current levels of experienced stress.[95] Items are scored on a scale from 0-4 with higher scores reflecting more perceived stress. This measure has been shown to be valid and internally consistent, with an alpha of .83.[94]

Elkins Hypnotizability Scale (EHS)

The EHS is a 12-item, research assistant-administered, scale for rating hypnotizability in the general population. Each item is rated pass\fail, with higher scores indicative of greater hypnotizability.

Previous research indicates that the EHS is highly correlated with longer measures of hypnotizability and has excellent reliability with a Cronbach's alpha of .94.[96]

Self-Hypnosis Practice Log

Frequency of home practice is thought to be one of the moderators of the efficacy of hypnosis interventions[70] and will be particularly important to collect for this self-administered protocol.[70] Participants in both groups will be given a log that they will complete daily. The log will provide space to put the date, whether or not the participant listened to their audio recording, the number of times they practiced (with and without the audio recordings), and provide a space for comments about any interruptions or barriers to listening to their assigned recording. Between the log and the phone calls, the research assistant will be able to ascertain the degree to which women adhered to the protocol in terms of recording hot flashes in real time and using the audio-recordings as prescribed in the protocol.

9.7 Data Analyses

In general, we will conduct an intent-to-treat (ITT) analysis in which all participants randomized to a treatment condition will be analyzed as such regardless of noncompliance, withdrawal, etc. A common strategy for ITT analysis when participants withdraw from a study is to carry the last observation forward. Given the likely underestimation of the variability due to that approach, we will employ multiple imputation to address missing data. We will also analyze the data based on completers and for those who have primary endpoint data at 6 weeks. We will explore the differences in the outcomes; and also explore which characteristics are present in those who do and do not have primary endpoint data. (See section 9.6, missing data).

Objective #1

The primary outcome will be change in hot flash scores over time during the treatment weeks (Weeks 1 – 6) compared to baseline between groups. Seven hot flash scores will be used (i.e., baseline score + weekly score for weeks 1 through 6) for each group. Hot flash scores will be used as the dependent variable in a longitudinal regression model that accounts for both group assignment as well as changes across the seven measurement points. The effective number of observations for this model is 1,624 observations (232 participants × 7 hot flash scores = 1,624). A second model will be constructed to include the week 12 (follow-up) data, which is an 8th observation for each participant to evaluate treatment sustainability as a secondary outcome. The effective number of observations for the model is 1,856 observations (232 participants × 8 hot flash scores = 1,856). The effect of the hypnosis intervention will be determined in these models by the difference between the changes hot flash scores (i.e., slopes) between the hypnosis and control groups. Important potential confounder variables (e.g., race/ethnicity, education, etc.) can easily be included in these models to account for their effects. The model described above is shown algebraically below.

Level 1 Model:
Level 1 Model:

[illegible]$_{ti} = \pi_{0i} + \pi_{1i} a_{ti} + e_{ti}$

[illegible]$_{ti}$ = Hot flash score at time t for participant i
π_{0i} = Intercept (average) hot flash score at baseline
π_{1i} = Amount of change in hot flash score associated with time t
a_{ti} = Value of time t

e_{ti} = Random error in hot flash score at time t for participant i $[e_{ti} \sim N(0, \sigma^2)]$

Level 2 Model:

$\pi_{0i} = \beta_{00} + \beta_{01}$ [illegible] $+ r_{0i}$

$\pi_{1i} = \beta_{10} + \beta_{11}$�$_1 + r_{1i}$

β_{00} = Grand mean hot flash score at baseline for control group
β_{01} = Expected difference in hot flash score at baseline between treatment and control groups
� = Value of Treatment indicator (X); 0 = control, 1 = treatment
β_{10} = Average amount of change in hot flash score across time for control group

β_{11} = Difference in amount of change in hot flash score across time between treatment and control group
r_{0i} = Random error of intercept for participant i adjusted from group assignment [$r_{0i} \sim N(0, \tau_{00})$]
r_{1i} = Random error of amount of change for participant i adjusted from group assignment [)]

[$r_{1i} \sim N(0, \tau_{10})$]

Combined (Mixed) Model:

�$= \beta_{00} + \beta_{01}$�$+ \beta_{10}$�$+ \beta_{11}$�$a_{ti} + r_{1i}a_{ti} + r_{0i} + e_{ti}$

The effect of primary interest is the interaction between time and treatment, which is the

β_{11}�a_{ti} term.

Objective #2

Secondary outcomes (activity interference from hot flashes, sleep disturbance, stress, and anxiety) will be entered separately as dependent variables into a similar type of longitudinal regression model described above. The primary difference in models is that for the secondary objectives, one set of models will be estimated using data collected at two points (i.e., baseline & week 6) and another set of models will be estimated using data collected at three time points (i.e., baseline, week 6, & week 12 [follow-up]).

Objective #3

The focus of Aim #3 is to determine the extent to which cortisol levels, HRV, and perceived stress mediate the relationship between group assignment and hot flashes. For each, we will test the null hypothesis that there is no mediation, or indirect effect, of these potential mediators on the hot flashes although we hypothesize that the relationship between group assignment and hot flashes will be mediated by these variables. Measurement considerations are presented for cortisol levels and HRV followed by a description of the mediation analysis plan. Cortisol levels will be evaluated using a commercial immunoassay with chemiluminescence detection by the CLASS lab at the University of Michigan for all samples from Baylor and Michigan. The lower concentration limits are 0.44 nmol/liter with intra- and interassay coefficients less than 8%. Cortisol slopes will be determined by recording cortisol values at three different times of day for each of the two collection days at baseline and at the end of the intervention, week 6. Higher slope values will reflect flatter diurnal cortisol rhythms. If values are found to be non-normal, we will use log transformations.[99] For HRV, we will extract beat-to-beat (NN) intervals from the recordings collected by the Polar S810. From these, we will use the well-validated Kubios HRV analysis software package[100] to calculate time domain indices of parasympathetic activity (standard deviation of NN intervals, SDNN; and root mean square of standard deviation of NN intervals, RMSSD) according to standard guideline.[101] These measures cover both short and longer term recordings and will be collected at baseline and the end of the intervention, week 6.

Mediation analysis will be performed using a nonparametric bootstrap method, to determine how much of the improvement in the primary and secondary outcomes is mediated by

psychophysiological effects of the intervention and by changes in perceived stress. The nonparametric bootstrap method provides more statistical power than traditional mediation analysis and does not require the assumption of normality. Within-subject change in HRV, cortisol slopes and perceived stress scores will be tested as mediators. Average causal mediation effects (ACME) and average direct effects (ADE) will be calculated to compare the mediated and un-mediated portion of the changes in the primary and secondary outcomes between groups. We will also test if pre-trial HRV predicts treatment effects, as we expect that women with high SNS activity at baseline will have the most to gain.

Moderator analysis will be performed using the longitudinal regression models described above. The benefit of using the longitudinal regression model for examining moderation is that the unique contribution of each moderator to the variability between people/groups or to the variability across time can be determined.

Missing Data

Based upon our previous research (R34 AT008246-03) we anticipate minimal missing data on the primary outcome (hot flash scores) and drop-outs of 18% or less of those that start treatment; and the specific pattern of missing data found in our prior study was primarily toward the end of data collection. Therefore, in the present study some missing data is anticipated, particularly at follow-up (week 12). Our statistician has advanced experienced in R, Mplus, and SAS packages and has focused training in missing data analysis in Mplus and SAS. To handle missing data we will follow Little's (1995) recommendation by collecting multiple pieces of evidence for the tenability of missing completely at random (MCAR) assumption if we experience substantial (e.g., > 5%) missingness. That is, as appropriate we will (a) conduct Little's (1988) test of missing completely at random using the observed data as input, (b) test the equality of the distributions of each of the outcome measures with missing data patterns using t test for location, (c) compute the standardized mean difference in each of the outcome measures across missing data patterns, and (d) examine the distribution of demographic variables across missing data patterns. If the possibility of systematic bias is present, we will most likely consider a multiple imputation method using Mplus or SAS. Again, our statistician has experience and coding knowledge to conduct the analyses in either package. Additionally, we will collect multiple pieces of information that allow us to assess the extent to which excluding those who did not complete the entire protocol may introduce systematic bias. We note that missing data in longitudinal data collection studies has been referred to by Little and Rubin[102] and Schafer and Graham[103] as "monotone" because the missingness tends to appear toward the end of data collection rather than the beginning (as we anticipate based upon our prior studies). When the attrition mechanism does not depend on observed or unobserved outcome variables, the pattern can be described as completely random drop-out[104] which is a special case of Rubin's[105] MCAR assumption.[106] We acknowledge that a standard mixed-model analysis or the use of HLM package will not handle different patterns of missing data on the six weeks of hot flash diaries or home practice logs. As noted, our statistician has experience and coding knowledge to conduct analysis in R, Mplus, and SAS packages and has focused training in missing data analysis in Mplus and SAS.

10. DATA COLLECTION AND QUALITY ASSURANCE

10.1 Data Collection Forms

Information is provided by participants through written responses to questionnaires, and through physiological measures of cortisol (saliva) and heart rate variability. The self-report measures will be assessed at baseline, week 6, and week 12, with the exception of the Hot Flash Daily Diary which

will be completed daily from baseline through week 6, and for one week at week 12. The Self-Hypnosis Practice Log will be completed daily during weeks 1 through 6 of the intervention and again during week 12. Physiologic measures will be collected at baseline and week 6.

Baseline questionnaires will be completed in the lab during the baseline interaction. During the COVID-19 restrictions, the questionnaires will be mailed to the participant with postage-paid envelope for return or sent electronically. The baseline Hot Flash Daily Diary, cortisol collection, and HRV data will be collected at home during the 7 days following the baseline interaction. The baseline hot flash diary, saliva kits and HRV monitor will be given to the participant at the baseline interaction (or mailed to the participant during the COVID-19 restrictions) with comprehensive education about the data collection. The HRV monitor will be worn for the designated time during the two days of cortisol collection. A second interaction within 30 days of the baseline data collection will be scheduled and serve as the randomization/education interaction. At this visit, participants will be given a Toolkit which will include:

- Intervention materials
  - weekly education/instruction booklets and audio recordings
- Data collection forms
  - Hot Flash Daily Diaries
  - Self-Hypnosis Practice Log
- Questionnaire booklets
  - a compilation of all outcome questionnaires for data collection at Weeks 6 and 12
- Return-addressed mailers (*for the return of study materials and data forms)*

In the event that the randomization/education visit must take place through telephone or video conference due to COVID-19 restrictions, the Toolkit, containing the appropriate intervention materials, will be mailed or sent electronically to the participant prior to the randomization/education visit. Then, the participant will have these materials prior to the randomization/education visit, and these materials can be discussed in detail during the interaction.

The baseline Hot Flash Daily Diary, saliva, and the HRV monitor will be mailed to the lab at the end of the baseline week. These materials can also be submitted electronically if the randomization/education visit takes place by telephone or video conference during the COVID-19 restrictions. If the visit takes place in person (at a time when COVID-19 restrictions are not in place), it may be brought to the lab at the time of the randomization/education visit. Staff receiving the data will be blinded to group assignment of the participant returning the data collection materials, and data will be entered upon receipt by a blinded data collector.

Before week 6, a package will be mailed to the participant with cortisol collection materials and an HRV monitor. At the end of Week 6, participants will complete the outcome measures in the Questionnaire Booklet, a second 2-day cortisol collection and wear the HRV for the designated time at some point during the two-day cortisol collection period. Once the questionnaires and physiological data collection is complete, participants will return to the lab in a pre-paid mailer:

- Six, week-long Hot Flash Daily Diaries
- The Self-Hypnosis Practice Log
- Questionnaires Booklet
- Cortisol collection materials
- HRV monitor

However, if this collection was optional or unable to be completed due to COVID-19 restrictions, saliva samples and HRV data will not be collected at this time.

During Week 11, a phone call will be made to participants to remind them of the 12 week data collection which includes a 7-day Hot Flash Daily Diary, 7-Day Self-Hypnosis Practice Log, and self-report questionnaires. This will all be in the toolkit with a pre-paid mailer. At the end of the Week 12, participants will complete the questionnaires and return the Hot Flash Daily Diary, Self-Hypnosis Practice Log, and Questionnaire booklet.

Data collectors, individuals responsible for data entry, PIs, and the study statistician will be blinded to the participants' group assignment, but the research staff who will randomize and research assistants who will call participants will be aware of the participant's group assignment. Participants will also be blinded to the study hypotheses to avoid bias regarding what is the experimental treatment versus control arm.

All materials collected are for research purposes only, and data will be kept in strict confidence. No information will be given to anyone without permission from the participant. The consent form includes the informed consent statement required by NCCIH and Baylor University and University of Michigan IRBs for studies involving human subjects. This statement guarantees confidentiality and identifies the subject as the owner of the information received. Several methods will be used to protect participant confidentiality. Identifying information is separated from all data provided by participants through the use of randomly generated study identification code assigned to each participant. Identifying information that is collected on paper will be kept in a closed envelope in a locked file cabinet separate from the participant charts.

Only authorized research staff will have keys to the project file room.

In addition, all personnel will be trained in the importance of and measures to protect participant confidentiality according to Good Clinical Practice and ICH Guidelines. Signed confidentiality statements will be on file for every research staff member.

Analyses will include only summaries of data, and personal identifiers will be excluded.

All research staff involved in the design or conduct of this research will have demonstrated successful completion and certification in Human Subjects Protection Education.

10.2 Data Management

Data will be collected at two clinical sites: Baylor University and University of Michigan, and both sites will have the same clinical site responsibilities (See *10.1 Data Collection Forms*) regarding data collection and management, as each site will be responsible for the data collection of 116 participants. No HIPPAA protected data will be shared between sites.

Management of HRV data: The staff receiving the HRV watches at Baylor/Michigan will download the data directly to the Kubios HRV analysis program and perform a series of automated pre-processing steps, then send the Kubios files to Dr. Tierney Lorenz for review and quality assurance, and final preparation for analysis. No HIPPAA protected data will be associated with any data from the HRV device. A HIPAA compliant, encrypted, password protected shared site, such as Mbox (dropbox) will be used. Kubios has just put out a "premium" version, which adds support for the direct transfer of files from Polar watches (rather than extracting by hand) and automated pre-processing options. This premium version will be purchased and used. Dr. Lorenz will provide comprehensive training to Baylor and University of Michigan research staff and will oversee all analysis and procedures.

As the Coordinating Center, Baylor University assumes the following responsibilities:

- Coordinate decisions regarding the design and development of the protocol and informed

consent documents for use at Baylor University and University of Michigan

- Review and approve all documents used at the two sites
- Ascertain that the protocol is reviewed and approved by the IRB at each institution prior to enrollment of participants at that site
- Ensure that each collaborating institution holds an applicable OHRP approved Federal Wide Assurance (FWA)
- Collect and maintain critical documents from affiliated investigators, (e.g. resume/CV, licenses, certification of completion of training)
- Store and/or manage data, data analysis, and data and safety monitoring activities. This will also be done at University of Michigan and reported to Baylor as per the DSMB document.
- Provide a study specific manual for phone contact and interaction with participants by research assistants and staff at both sites.
- Develop and provide protocol specific case report forms as needed for both sites.
- Coordinate randomization.
- Track participant enrollment and provide enrollment reports to NCCIH every 4 months.
- Ensure that both sites are using the correct version of the protocol and consent document.
- Ensure that both sites are utilizing quality control measures to assure data accuracy and completeness.
- Track, report and maintain documentation of all serious adverse events and unanticipated problems and disseminating the information to both sites.
- Share periodic updates between investigators on participant enrollment, general study progress, and relevant scientific advances.
- Assure that all relevant IRB correspondence (continuing review and amendments) and study status changes are communicated at both sites.

10.3 Quality Assurance

10.3.1 Training

Training of the research personnel for both arms at both locations will involve role playing and the use of scripts to ensure the appropriate delivery of the protocol, communication with participants, collection of measures, and assessment of symptoms and progress. This applies to both treatment arms. This training will be conducted by both Dr. Barton and Dr. Elkins in person with both research teams to ensure consistency of information and training. Throughout the study, Drs. Elkins and Barton will perform fidelity checks on study procedures at their respective institutions.

10.3.2 Quality Control Committee

Not applicable

10.3.3 Metrics

Not applicable

10.3.4 Protocol Deviations

Procedures for protocol deviations will be as follows:

- All protocol deviations will be recorded on the Protocol Deviation Tracking Log and reported within 48 hours after their identification to the PI

- PI will then report deviations to other members of the investigative team as necessary
- Deviations involving informed consent or risk to study participants will be reported to the site local IRB within 48 hours of their identification by site PI or designee.
- Deviations will be recorded on the DSMB report and reported to NCCIH with corrective action every 6 months and yearly to IRB as mandated.

Any missing data regarding uncollected saliva samples or unrecorded HRV readings by participants will be considered missed data and not protocol deviations. This also applies to when participants are unable to have the EHS-CF administered via remote procedures during COVID-19 restrictions. Missing data from the participant declining the option or inability of being administered the EHS-CF will be treated as missed data and not protocol deviations.

10.3.5 Monitoring

NCCIH has an on-site monitoring program and this study will be visited by an NCCIH-designated study monitor prior to participant enrollment, approximately mid-way through the enrollment phase, and at study completion. During these site visits, the materials to be reviewed include, but may not be limited to:

- Participant ICFs
- All source documents
- Case Report Forms
- Data Entry database
- All regulatory and other pertinent documents (e.g., IRB approvals, consent documents, etc.)
- Delegation of Authority Log
- Research Staff Training documentation
- Adverse Event documentation
- Reporting documents (e.g., DSMB, NCCIH Progress Reports, IRB Annual Review)
- Manual of Procedures

11. PARTICIPANT RIGHTS AND CONFIDENTIALITY

11.1 Institutional Review Board (IRB) Review

This protocol and the informed consent form (ICF) and any subsequent modifications will be reviewed and approved by the IRBs at the respective site institutions (Baylor University or University of Michigan). The consent form should be separate from the protocol document.

11.2 Informed Consent Forms

There will be separate consent forms for Baylor and for the University of Michigan. The informed consent will provide documentation of the research study including: 1) Purpose and Background, 2) Procedures, 3) Discomfort and Risks, 4) Benefits, 5) Cost and Compensation, 6) Right to Withdraw Consent and Authorization, 7) Confidentiality, 8) Right to Access Study Information, 9) Contact for Questions or Emergencies, and 10) Statement of Consent and Authorization/Statement of Person Obtaining Consent. Participants consenting to participation will sign the IRB-approved consent

form which will also be signed by the CRC or a member of the research staff. All participants will be given an opportunity to ask questions and have the overall objectives of the study explained in easily understandable terms by the PI, CRC, or research staff.

Each participant will be given a copy of the consent form and the original will be filed in a folder identified with the participant's unique study identifier. This document will be kept separate from all case report forms or other study materials.

11.3 Participant Confidentiality

The participants' safety and confidentiality of the data are maintained at the highest level possible. Any information about participants obtained from the proposed research will be kept confidential. Participants will not be personally identified in any publication or presentation resulting from this study. The Data and Safety Monitoring Board (DSMB) will assess data confidentiality and safety throughout the study period. The Data and Safety Monitoring Board will be comprised of three individuals and is detailed in the DSMB document attached.

At each institution, identifying information is separated from all data provided by participants through the use of a randomly generated study identification code assigned to each participant. A code list will be maintained by the research coordinator at each site and stored on an encrypted file. Identifying information that is collected on paper will be kept in a closed envelope in a locked file cabinet separate from the participant charts or redacted. No identifiable data will be shared across institutions. In addition, only the PI and the project team will have keys to the project file room. Research staff will be trained to know the vital importance of protecting participant information and signed confidentiality statements for all research staff will be kept on file. Analyses will include only summaries of data, and personal identifiers will be excluded. All research staff involved in the design or conduct of this research will have demonstrated successful completion and certification in Human Subjects Protection.

11.4 Study Discontinuation

This study may be discontinued at any time by the IRB, the NCCIH, the OHRP, or other government agencies as part of their duties to ensure that research participants are protected.

12. COMMITTEES

Not applicable

13. PUBLICATION OF RESEARCH FINDINGS

This research is supported by an NIH award, and therefore any publication, press release, or other document about this research will include an acknowledgment of NIH award support and a disclaimer such as "Research reported in this publication was supported by the National Center for Complementary & Integrative Health of the National Institutes of Health under Award Number R01AT009384. The content is solely the responsibility of the authors and does not necessarily represent the official views of the National Institutes of Health."

14. REFERENCES

1. NAMS, ed. *Menopause Practice: A Clinicians Guide* 5th ed. 2014, North American Menopause Society: OH.
2. Stearns, V., et al., *Hot flushes.* The Lancet, 2002. 360(9348): p. 1851-1861.
3. Gold, E.B., et al., *Longitudinal analysis of the association between vasomotor symptoms and race/ethnicity across the menopausal transition: study of women's health across the nation.* American journal of public health, 2006. 96(7): p. 1226-1235.
4. Avis, N.E., et al., *Duration of menopausal vasomotor symptoms over the menopause transition.* JAMA internal medicine, 2015. 175(4): p. 531-539.
5. Gupta, P., et al., *Menopausal symptoms in women treated for breast cancer: the prevalence and severity of symptoms and their perceived effects on quality of life.* Climacteric, 2006. 9(1): p. 49-58.
6. Davis, S.R., et al., *Menopausal symptoms in breast cancer survivors nearly 6 years after diagnosis.* Menopause, 2014. 21(10): p. 1075-1081.
7. Dennerstein, L., et al., *New findings from non-linear longitudinal modelling of menopausal hormone changes.* Human Reproduction Update, 2007. 13(6): p. 551-557.
8. Fisher, W.I., et al., *Risk factors, pathophysiology, and treatment of hot flashes in cancer.* CA: a cancer journal for clinicians, 2013. 63(3): p. 167-192.
9. Carpenter, J.S., et al., *Hot flashes in postmenopausal women treated for breast carcinoma.* Cancer, 1998. 82(9): p. 1682-1691.
10. Kronenberg, F., *Hot flashes: phenomenology, quality of life, and search for treatment options.* Experimental gerontology, 1994. 29(3): p. 319-336.
11. Patel, S.R., et al., *A prospective study of sleep duration and mortality risk in women.* Sleep, 2004. 27(3): p. 440-444.
12. Hublin, C., et al., *Sleep and mortality: a population-based 22-year follow-up study.* Sleep, 2007. 30(10): p. 1245-1253.
13. Williams, R.E., et al., *Menopause-specific questionnaire assessment in US population-based study shows negative impact on health-related quality of life.* Maturitas, 2009. 62(2): p. 153-159.
14. Stein, K.D., et al., *Impact of hot flashes on quality of life among postmenopausal women being treated for breast cancer.* Journal of pain and symptom management, 2000. 19(6): p. 436-445.
15. Kronenberg, F., *Hot flashes: Epidemiology and physiologya.* Annals of the New York Academy of Sciences, 1990. 592(1): p. 52-86.
16. Thurston, R.C., et al., *Association between hot flashes, sleep complaints, and psychological functioning among healthy menopausal women.* International journal of behavioral medicine, 2006. 13(2): p. 163-172.
17. Investigators, W.G.f.t.W.s.H.I., *Risks and benefits of estrogen plus progestin in healthy postmenopausal women: principal results From the Women's Health Initiative randomized controlled trial.* Jama, 2002. 288(3): p. 321-333.
18. Loprinzi, C.L., et al., *Megestrol acetate for the prevention of hot flashes.* New England Journal of Medicine, 1994. 331(6): p. 347-352.
19. Society, N.A.M., *The 2012 hormone therapy position statement of the North American Menopause Society.* Menopause (New York, NY), 2012. 19(3): p. 257.
20. Holmberg, L., et al., *Increased risk of recurrence after hormone replacement therapy in breast cancer survivors.* Journal of the National Cancer Institute, 2008. 100(7): p. 475-482.
21. Couzi, R.J., K.J. Helzlsouer, and J.H. Fetting, *Prevalence of menopausal symptoms among women with a history of breast cancer and attitudes toward estrogen replacement therapy.* Journal of Clinical Oncology, 1995. 13(11): p. 2737-2744.
22. Rada, G., et al., *Non-hormonal interventions for hot flushes in women with a history of breast cancer.* Sao Paulo Medical Journal, 2013. 131(2): p. 141-141.

23. Nachtigall, L.E., *Therapy: Nonhormonal treatment of hot flashes—a viable alternative?* Nature Reviews Endocrinology, 2010. 6(2): p. 66-67.
24. Loprinzi, C.L., et al., *Newer antidepressants and gabapentin for hot flashes: an individual patient pooled analysis.* Journal of Clinical Oncology, 2009. 27(17): p. 2831-2837.
25. Ramaswami, R., et al., *Venlafaxine in management of hot flashes in women with breast cancer: a systematic review and meta-analysis.* Breast cancer research and treatment, 2015. 152(2): p. 231-237.
26. Nelson, H.D., et al., *Nonhormonal therapies for menopausal hot flashes: systematic review and meta-analysis.* Jama, 2006. 295(17): p. 2057-2071.
27. Wiśniewska, I., et al., *The pharmacological and hormonal therapy of hot flushes in breast cancer survivors.* Breast Cancer, 2016. 23(2): p. 178-182.
28. *Gabapentin Package Insert*. 19 April 2017;]. Available from: http://medlibrary.org/lib/rx/meds/neurontin-3/.
29. Avis, N.E., *Breast cancer survivors and hot flashes: the search for nonhormonal treatments.* 2008, American Society of Clinical Oncology.
30. Hickey, M., C.M. Saunders, and B.G. Stuckey, *Management of menopausal symptoms in patients with breast cancer: an evidence-based approach.* The lancet oncology, 2005. 6(9): p. 687-695.
31. Carmody, J., S. Crawford, and L. Churchill, *A pilot study of mindfulness-based stress reduction for hot flashes.* [illegible]nopause, 2006. 13(5): p. 760-769.
32. Hunter, M.S., et al., *Menopausal sympto[illegible]n women with breast cancer: prevalence and treatment preferences.* Psycho- Oncology, 2004. 13(11): p. 769-778.
33. Ayers, B., et al., *Effectiveness of group and self-help cognitive behavior therapy in reducing problematic menopausal hot flushes and night sweats (MENOS 2): a randomized controlled trial.* Menopause, 2012. 19(7): p. 749-759.
34. Mann, E., et al., *Cognitive behavioural treatment for women who have menopausal symptoms after breast cancer treatment (MENOS 1): a randomised controlled trial.* The lancet oncology, 2012. 13(3): p. 309-318.
35. Barton, D.L. and C.L. Loprinzi, *Using one's head to treat menopausal symptoms*. 2012, American Society of Clinical Oncology.
36. Sood, R., et al., *Paced breathing compared with usual breathing for hot flashes.* Menopause, 2013. 20(2): p. 179-184.
37. Sloan, J.A., et al., *Methodologic lessons learned from hot flash studies.* Journal of Clinical Oncology, 2001. 19(23): p. 4280-4290.
38. Elkins, G., et al., *Randomized trial of a hypnosis intervention for treatment of hot flashes among breast cancer survivors.* Journal of Clinical Oncology, 2008. 26(31): p. 5022-5026.
39. Elkins, G.R., et al., *Clinical hypnosis in the treatment of post-menopausal hot flashes: a randomized controlled trial.* Menopause (New York, NY), 2013. 20(3).
40. David, S.M., et al., *Randomised controlled trial comparing hypnotherapy versus gabapentin for the treatment of hot flashes in breast cancer survivors: a pilot study.* BMJ open, 2013. 3(9): p. e003138.
41. Barton, D., et al. *Pilot study of a biobehavioral treatment for hot flashes*. in *ANNALS OF BEHAVIORAL MEDICINE*. 2013. SPRINGER 233 SPRING ST, NEW YORK, NY 10013 USA.
42. Carpenter, J., et al., *Nonhormonal management of menopause-associated vasomotor symptoms: 2015 position statement of The North American Menopause Society.* Menopause, 2015. 22(11): p. 1155-1174.
43. Council, N.R., *Women's health research: progress, pitfalls, and promise*. 2010, Washington, DC: The National Academies Press.
44. Appt, S.E. and K.F. Ethun, *Reproductive aging and risk for chronic disease: Insights from studies of nonhuman primates.* Maturitas, 2010. 67(1): p. 7-14.
45. Barton, D.L. and P.A. Ganz, *Symptoms: menopause, infertility, and sexual health*, in *Improving Outcomes for Breast Cancer Survivors*. 2015, Springer. p. 115-141.

46. Hamunen, K., et al., *Effect of pain on autonomic nervous system indices derived from photoplethysmography in healthy volunteers.* British journal of anaesthesia, 2012. 108(5): p. 838-844.
47. Terkelsen, A.J., et al., *Cutaneous noradrenaline measured by microdialysis in complex regional pain syndrome during whole-body cooling and heating.* Experimental neurology, 2013. 247: p. 456-465.
48. Breimhorst, M., et al., *Sensory and sympathetic correlates of heat pain sensitization and habituation in men and women.* European Journal of Pain, 2012. 16(9): p. 1281-1292.
49. Loggia, M.L., M. Juneau, and M.C. Bushnell, *Autonomic responses to heat pain: Heart rate, skin conductance, and their relation to verbal ratings and stimulus intensity.* PAIN®, 2011. 152(3): p. 592-598.
50. Koenig, J., et al., *Parasympathetic Activity in Young Females Related to Pain Tolerance but not to Pain Threshold in Induced Thermal Pain.* Age (years), 2012. 31(20.52): p. 2.99.
51. Nahman-Averbuch, H., et al., *Sex differences in the relationships between parasympathetic activity and pain modulation.* Physiology & behavior, 2016. 154: p. 40-48.
52. Kekecs, Z., A. Szekely, and K. Varga, *Alterations in electrodermal activity and cardiac parasympathetic tone during hypnosis.* Psychophysiology, 2016. 53(2): p. 268-277.
53. Debenedittis, G., et al., *Autonomic changes during hypnosis: a heart rate variability power spectrum analysis as a marker of sympatho-vagal balance.* International Journal of Clinical and Experimental Hypnosis, 1994. 42(2): p. 140-152.
54. de Zambotti, M., et al., *Vagal withdrawal during hot flashes occurring in undisturbed sleep: Hot flashes and autonomic activity.* Menopause (New York, NY), 2013. 20(11).
55. Gerber, L.M., et al., *Hot flashes are associated with increased ambulatory systolic blood pressure.* Menopause, 2007. 14(2): p. 308-315.
56. Morrow, P.K.H., D.N. Mattair, and G.N. Hortobagyi, *Hot flashes: a review of pathophysiology and treatment modalities.* The oncologist, 2011. 16(11): p. 1658-1664.
57. Swartzman, L.C., R. Edelberg, and E. Kemmann, *Impact of stress on objectively recorded menopausal hot flushes and on flush report bias.* Health Psychology, 1990. 9(5): p. 529.
58. Gannon, L., S. Hansel, and J. Goodwin, *Correlates of menopausal hot flashes.* Journal of Behavioral Medicine, 1987. 10(3): p. 277-285.
59. Walega, D.R., et al., *Effects of stellate ganglion block on vasomotor symptoms: findings from a randomized, controlled clinical trial in postmenopausal women.* Menopause (New York, NY), 2014. 21(8): p. 807.
60. Gastel, P.v., et al., *Stellate-ganglion block as a treatment for severe postmenopausal flushing.* 2013.
61. Lee, M.S., et al., *Acupuncture for treating hot flashes in breast cancer patients: a systematic review.* Breast cancer research and treatment, 2009. 115(3): p. 497.
62. Cramer, H., et al., *Effectiveness of yoga for menopausal symptoms: a systematic review and meta-analysis of randomized controlled trials.* Evidence-Based Complementary and Alternative Medicine, 2012. 2012.
63. Aubert, A.E., et al., *Cardiac autonomic regulation under hypnosis assessed by heart rate variability: spectral analysis and fractal complexity.* Neuropsychobiology, 2009. 60(2): p. 104-112.
64. v Hippel, C., G. Hole, and W. Kaschka, *Autonomic profile under hypnosis as assessed by heart rate variability and spectral analysis.* Pharmacopsychiatry, 2001. 34(03): p. 111-113.
65. Camm, A.J., et al., *Heart rate variability: standards of measurement, physiological interpretation and clinical use. Task Force of the European Society of Cardiology and the North American Society of Pacing and Electrophysiology.* Circulation, 1996. 93(5): p. 1043-1065.
66. Sassi, R., et al., *Advances in heart rate variability signal analysis: joint position statement by the e-Cardiology ESC Working Group and the European Heart Rhythm Association co-endorsed by the Asia Pacific Heart Rhythm Society.* EP Europace, 2015. 17(9): p. 1341-1353.
67. Sephton, S.E., et al., *Diurnal cortisol rhythm as a predictor of breast cancer survival.* Journal of the National Cancer Institute, 2000. 92(12): p. 994-1000.

68. Kirschbaum, C. and D.H. Hellhammer, *Salivary cortisol in psychoneuroendocrine research: recent developments and applications.* Psychoneuroendocrinology, 1994. 19(4): p. 313-333.
69. Elkins, G., et al., *Preferences for hypnotic imagery for hot-flash reduction: a brief communication.* Intl. Journal of Clinical and Experimental Hypnosis, 2010. 58(3): p. 345-349.
70. Jensen, M. and D.R. Patterson, *Hypnotic treatment of chronic pain.* Journal of behavioral medicine, 2006. 29(1): p. 95-124.
71. Freeman, E.W., et al., *The role of anxiety and hormonal changes in menopausal hotflashes.* Menopause, 2005. 12(3): p. 258-266.
72. McCorry, L.K., *Physiology of the autonomic nervous system.* American journal of pharmaceutical education, 2007. 71(4): p. 78.
73. Elkins, G., et al., *A pilot investigation of guided self-hypnosis in the treatment of hot flashes among postmenopausal women.* International Journal of Clinical and Experimental Hypnosis, 2013. 61(3): p. 342-350.
74. Freeman, E.W., et al., *Placebo improvement in pharmacologic treatment of menopausal hot flashes: time course, duration, and predictors.* Psychosomatic medicine, 2015. 77(2): p. 167.
75. Loprinzi, C.L., et al., *Mayo Clinic and North Central Cancer Treatment Group hot flash studies: a 20-year experience.* Menopause, 2008. 15(4): p. 655-660.
76. Lorenz, T.A., et al., *Evidence for a curvilinear relationship between sympathetic nervous system activation and women's physiological sexual arousal.* Psychophysiology, 2012. 49(1): p. 111-117.
77. Stanton, A.M., et al., *Heart rate variability: A risk factor for female sexual dysfunction.* Applied psychophysiology and biofeedback, 2015. 40(3): p. 229-237.
78. Lorenz, T.K., C.B. Harte, and C.M. Meston, *Changes in autonomic nervous system activity are associated with changes in sexual function in women with a history of childhood sexual abuse.* The journal of sexual medicine, 2015. 12(7): p. 1545-1554.
79. Meston, C.M. and T.A. Lorenz, *Physiological stress responses predict sexual functioning and satisfaction differently in women who have and have not been sexually abused in childhood.* Psychological trauma: theory, research, practice, and policy, 2013. 5(4): p. 350.
80. Kroenke, K., et al., *An ultra-brief screening scale for anxiety and depression: the PHQ–4.* Psychosomatics, 2009. 50(6): p. 613-621.
81. Löwe, B., et al., *A 4-item measure of depression and anxiety: validation and standardization of the Patient Health Questionnaire-4 (PHQ-4) in the general population.* Journal of affective disorders, 2010. 122(1): p. 86-95.
82. Carpenter, J.S., *The Hot Flash Related Daily Interference Scale: a tool for assessing the impact of hot flashes on quality of life following breast cancer.* Journal of pain and symptom management, 2001. 22(6): p. 979-989.
83. Pilkonis, P.A., et al., *Item banks for measuring emotional distress from the Patient-Reported Outcomes Measurement Information System (PROMIS®): depression, anxiety, and anger.* Assessment, 2011. 18(3): p. 263-283.
84. Saffer, B.Y., et al., *Assessing cognitive impairment using PROMIS® applied cognition-abilities scales in a medical outpatient sample.* Psychiatry research, 2015. 226(1): p. 169-172.
85. Grandner, M.A., et al., *Criterion validity of the Pittsburgh Sleep Quality Index: Investigation in a non-clinical sample.* Sleep and Biological Rhythms, 2006. 4(2): p. 129-136.
86. Carpenter, J.S. and M.A. Andrykowski, *Psychometric evaluation of the Pittsburgh sleep quality index.* Journal of psychosomatic research, 1998. 45(1): p. 5-13.
87. Beaudreau, S.A., et al., *Validation of the Pittsburgh Sleep Quality Index and the Epworth Sleepiness Scale in older black and white women.* Sleep medicine, 2012. 13(1): p. 36-42.
88. Sloan, J., et al. *Clinical Significance Consensus Meeting Group. Assessing clinical significance in measuring oncology patient quality of life: introduction to the symposium, content overview, and definition of terms*. in *Mayo Clin Proc*. 2002.

89. Sloan, J.A., et al., *The clinical significance of quality of life assessments in oncology: a summary for clinicians.* Supportive Care in Cancer, 2006. 14(10): p. 988-998.
90. Hoikkala, H., et al., *Association between vasomotor hot flashes and heart rate variability in recently postmenopausal women.* Menopause, 2010. 17(2): p. 315-320.
91. Freedman, R.R., M.L. Kruger, and S.L. Wasson, *Heart rate variability in menopausal hot flashes during sleep.* Menopause (New York, NY), 2011. 18(8): p. 897.
92. Thurston, R.C., I.C. Christie, and K.A. Matthews, *Hot flashes and cardiac vagal control: a link to cardiovascular risk?* Menopause (New York, NY), 2010. 17(3): p. 456.
93. Giles, D., N. Draper, and W. Neil, *Validity of the Polar V800 heart rate monitor to measure RR intervals at rest.* European journal of applied physiology, 2016. 116(3): p. 563-571.
94. Lee, E.-H., *Review of the psychometric evidence of the perceived stress scale.* Asian Nursing Research, 2012. 6(4): p. 121-127.
95. Cohen, S., T. Kamarck, and R. Mermelstein, *A global measure of perceived stress.* Journal of health and social behavior, 1983: p. 385-396.
96. Elkins, G., *Hypnotic relaxation therapy: Principles and applications*. 2013: Springer Publishing Company.
97. Faul, F., et al., *G* Power 3: A flexible statistical power analysis program for the social, behavioral, and biomedical sciences.* Behavior research methods, 2007. 39(2): p. 175-191.
98. Spybrook, J., Bloom, H., Congdon, R., Hill, C., Liu, X., Martinez, A., and Raudenbush, S., *Optimal Design Plus Empirical Evidence*. 2013.
99. Keene, O.N., *The log transformation is special.* Stat Med, 1995. 14(8): p. 811-9.
100. Tarvainen, M.P., et al., *Kubios HRV--heart rate variability analysis software.* Comput Methods Programs Biomed, 2014. 113(1): p. 210-20.
101. *Heart rate variability. Standards of measurement, physiological interpretation, and clinical use. Task Force of the European Society of Cardiology and the North American Society of Pacing and Electrophysiology.* Eur Heart J, 1996. 17(3): p. 354-81.
102. Little, R.J. and D.B. Rubin, *Statistical analysis with missing data*. 2002: John Wiley & Sons.
103. Schafer, J.L. and J.W. Graham, *Missing data: our view of the state of the art.* Psychological methods, 2002. 7(2): p. 147.
104. Diggle, P. and M.G. Kenward, *Informative drop-out in longitudinal data analysis.* Applied statistics, 1994: p. 49-93.
105. Rubin, D.B., *Inference and missing data.* Biometrika, 1976. 63(3): p. 581-592.
106. Little, R.J., *Modeling the drop-out mechanism in repeated-measures studies.* Journal of the American Statistical Association, 1995. 90(431): p. 1112-1121.
