# Supplementary material for: Self-Administered Hypnosis vs Sham Hypnosis for Hot Flashes: A Randomized Clinical Trial
Source: JAMA Netw Open. 2025 Nov 11;8(11):e2542537. doi: 10.1001/jamanetworkopen.2025.42537 (PMC12606380; doi:10.1001/jamanetworkopen.2025.42537)
Supplement: Supplement 2. — eTable 1. Schedule of Study Activities eTable 2. Descriptive Statistics for Hot Flash Severity Frequency by Treatment Group eTable 3. Frequency Distributions (Percentages) of Reported Perceived Benefit of Intervention Across Groups eTable 4. Adverse Events eFigure. Profile Plots of Hot Flash Outcomes by Treatment Group (Self-Hypnosis vs. Sham Control) and Hot Flash Severity [file jamanetwopen-e2542537-s002.pdf]

Supplementary Online Content

Elkins G, Arring N, Morgan G, et al. Self-administered hypnosis vs sham hypnosis for hot flashes: a randomized clinical trial. *JAMA Netw Open.* 2025;8(11):e2542537. doi:10.1001/jamanetworkopen.2025.42537

eTable 1. Schedule of Study Activities
eTable 2. Descriptive Statistics for Hot Flash Severity Frequency by Treatment Group
eTable 3. Frequency Distributions (Percentages) of Reported Perceived Benefit of Intervention Across Groups
eTable 4. Adverse Events
eFigure. Profile Plots of Hot Flash Outcomes by Treatment Group (Self-Hypnosis vs. Sham Control) and Hot Flash Severity

This supplementary material has been provided by the authors to give readers additional information about their work.

© 2025 Elkins G et al. *JAMA Network Open.*

eTable 1. Schedule of Study Activities

	Enrollment	Baseline	Treatment and follow-up in weeks						
			**1**	**2**	**3**	**4**	**5**	**6**	**12**
Meeting with interventionist (virtual or in-person)	X	X							
Informed consent	X								
Demographic questionnaire & health history intake		X							
Hot flash daily diary		D	D	D	D	D	D	D	D
Hot flash related daily interference scale		X						X	X
PROMIS Emotional Distress- Anxiety Scale		X						X	X
Global impression of change								X	
Frequency of home practice		D	D	D	D	D	D	D	D
Check in phone calls			X	X	X	X	X	X	

Note. D = daily.

© 2025 Elkins G et al. *JAMA Network Open.*

eTable 2. Descriptive Statistics for Hot Flash Severity Frequency by Treatment Group

	Hypnosis Group						White Noise Group					
	Baseline		Week 6		Week 12		Baseline		Week 6		Week 12	
	M	SD	M	SD	M	SD	M	SD	M	SD	M	SD
Mild	21.0	16.1	16.7	13.2	14.7	12.9	19.1	14.6	16.2	17.6	13.0	12.2
Moderate	21.6	21.3	8.0	10.3	7.8	12.0	20.9	19.8	12.5	16.0	12.7	18.3
Severe	6.1	9.9	2.1	8.4	1.3	3.7	6.8	12.5	3.8	8.9	3.8	8.3
Very Severe	1.5	4.7	0.7	4.2	0.1	0.8	3.3	11.1	0.9	4.0	0.8	3.0

© 2025 Elkins G et al. *JAMA Network Open.*

eTable 3. Frequency Distributions (Percentages) of Reported Perceived Benefit of Intervention Across Groups

Magnitude of Change	Hypnosis Group	White Noise Group
Very much worse	0.0	0.0
Moderately worse	1.0	1.0
A little worse	1.9	4.1
About the same	6.8	30.6
A little better	17.5	25.5
Moderately better	40.8	20.4
Very much better	32.0	18.4

Note. $\chi^2_5 = 28.2$, $V = .38$, $p < .001$

© 2025 Elkins G et al. *JAMA Network Open.*

eTable 4. Adverse Events

<table>
<tr><th colspan="2"></th><th>Intervention</th><th>Control</th><th>Total</th></tr>
<tr><td colspan="2">Number of AEs Reported</td><td>7</td><td>8</td><td>15</td></tr>
<tr><td colspan="2">Number of Participants with AEs</td><td>4</td><td>5</td><td>9</td></tr>
<tr><td colspan="2">Number of Serious AEs Reported</td><td>0</td><td>0</td><td>0</td></tr>
<tr><td colspan="2">Number of Participants With Serious AEs</td><td>0</td><td>0</td><td>0</td></tr>
<tr><td colspan="2">Number of AEs by Intensity</td><td rowspan="2">7</td><td rowspan="2">8</td><td rowspan="2">15</td></tr>
<tr><td rowspan="4"></td><td>Mild</td></tr>
<tr><td>Moderate</td><td>0</td><td>0</td><td>0</td></tr>
<tr><td>Severe</td><td>0</td><td>0</td><td>0</td></tr>
<tr><td>Life Threatening/Disabling</td><td>0</td><td>0</td><td>0</td></tr>
<tr><td colspan="2">Number of AEs by Attribution</td><td rowspan="2">7</td><td rowspan="2">8</td><td rowspan="2">15</td></tr>
<tr><td rowspan="5"></td><td>Unrelated</td></tr>
<tr><td>Unlikely</td><td>0</td><td>0</td><td>0</td></tr>
<tr><td>Possible</td><td>0</td><td>0</td><td>0</td></tr>
<tr><td>Probable</td><td>0</td><td>0</td><td>0</td></tr>
<tr><td>Definite</td><td>0</td><td>0</td><td>0</td></tr>
</table>

Note. AE = Adverse Events

© 2025 Elkins G et al. *JAMA Network Open.*

eFigure. Profile Plots of Hot Flash Outcomes by Treatment Group (Self-Hypnosis vs. Sham Control) and Hot Flash Severity

S1a. Mild Hot Flashes

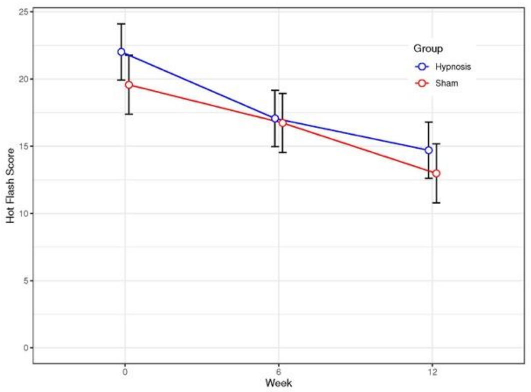

S1b. Moderate Hot Flashes

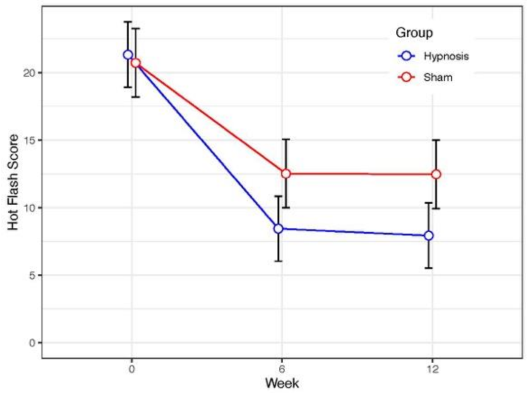

S1c. Severe Hot Flashes

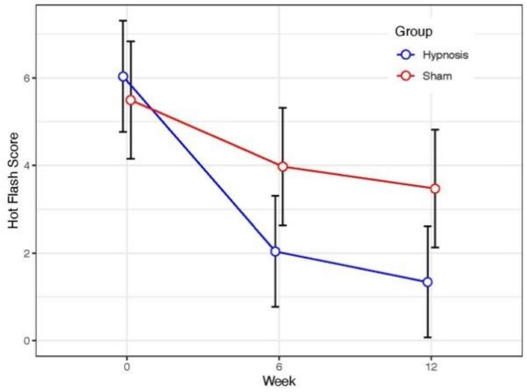

S1d. Very Severe Hot Flashes

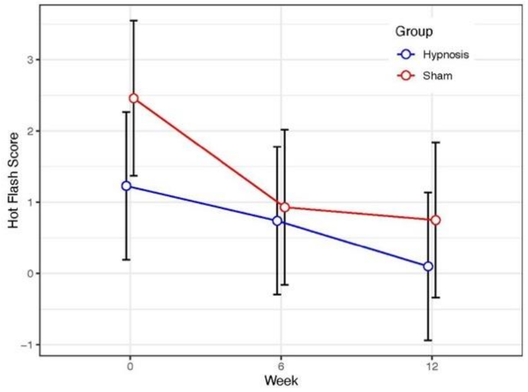

Across all hot flashes subsamples, the hot flash scores for both the self-hypnosis and sham hypnosis groups changed from baseline to Week 12, but the self-hypnosis group saw a greater decrease in hot flashes.

Note. Blue line represents the hypnosis group, and the red line represents the white noise group. Error bands indicate 95% confidence for the means at each timepoint during the study.

© 2025 Elkins G et al. *JAMA Network Open.*
