# Supplementary material for: Self-Administered Hypnosis vs Sham Hypnosis for Hot Flashes: A Randomized Clinical Trial
Source: JAMA Netw Open. 2025 Nov 11;8(11):e2542537. doi: 10.1001/jamanetworkopen.2025.42537 (PMC12606380; doi:10.1001/jamanetworkopen.2025.42537)
Supplement: Supplement 3. — Data Sharing Statement [file jamanetwopen-e2542537-s003.pdf]

Data Sharing Statement

Elkins. Self-Administered Hypnosis vs Sham Hypnosis for Hot Flashes. *JAMA Netw Open*. Published November 11, 2025. doi:10.1001/jamanetworkopen.2025.42537

Data
Additional Information: This study was registered at clinicaltrials.gov. The registration number was: NCT03572153, https://clinicaltrials.gov/study/NCT03572153?cond=Hot%20Flashes&term=Self%20Administration&intr=hypnosis&rank=1
Data available: Yes
Data types: Deidentified participant data
How to access data: Requests for deidentified participant data can be sent to gary_elkins@baylor.edu
When available: With publication

Supporting Documents
Document types: Statistical/analytic code, Informed consent form
How to access documents: Requests for statistical/analytic code and/or informed consent form can be sent to gary_elkins@baylor.edu
When available: With publication

Additional Information
Who can access the data: Data will be available to researchers whose request to the primary author gary_elkins@baylor.edu on use of data for research purposes has been approved
Types of analyses: Primary, Secondary and Pre-specified Exploratory Analyses will be made available. Any analysis proposal sent by a researcher looking to access other data shared will be reviewed by the study team.
Mechanisms of data availability: Deidentified data will be available with a data access agreement from the primary author after approval of the manuscript for submission and after all pre-specified exploratory and secondary analyses have been completed.
